# The Influence of Selected Antipsychotic Drugs on Biochemical Aspects of Alzheimer’s Disease

**DOI:** 10.3390/ijms23094621

**Published:** 2022-04-21

**Authors:** Maria Podsiedlik, Magdalena Markowicz-Piasecka, Joanna Sikora

**Affiliations:** 1 Department of Pharmaceutical Chemistry, Drug Analysis and Radiopharmacy, Medical University of Lodz, Muszynskiego 1, 90-151 Lodz, Poland; 2 Laboratory of Bioanalysis, Department of Pharmaceutical Chemistry, Drug Analysis and Radiopharmacy, Medical University of Lodz, Muszynskiego 1, 90-151 Lodz, Poland; magdalena.markowicz@umed.lodz.pl; 3 Department of Bioinorganic Chemistry, Medical University of Lodz, Muszynskiego 1, 90-151 Lodz, Poland; joanna.sikora@umed.lodz.pl

**Keywords:** Alzheimer’s disease, antipsychotic drugs, repurposing

## Abstract

The aim of this study was to assess the potency of selected antipsychotic drugs (haloperidol (HAL), bromperidol (BRMP), benperidol (BNP), penfluridol (PNF), pimozide (PIM), quetiapine (QUET) and promazine (PROM)) on the main pathological hallmarks of Alzheimer’s disease (AD). Binary mixtures of donepezil and antipsychotics produce an anti-BuChE effect, which was greater than either compound alone. The combination of rivastigmine and antipsychotic drugs (apart from PNF) enhanced AChE inhibition. The tested antipsychotics (excluding HAL and PNF) significantly reduce the early stage of Aβ aggregation. BRMP, PIM, QUET and PROM were found to substantially inhibit Aβ aggregation after a longer incubation time. A test of human erythrocytes hemolysis showed that short-term incubation of red blood cells (RBCs) with QUET resulted in decreased hemolysis. The antioxidative properties of antipsychotics were also proved in human umbilical vein endothelial cells (HUVEC); all tested drugs were found to significantly increase cell viability. In the case of astrocytes, BNP, PNF, PIM and PROM showed antioxidant potential.

## 1. Introduction

Alzheimer’s disease (AD), an irreversible and progressive disorder, is known for its heterogeneous etiology, but the exact cause of the disease remains unknown [1]. Current evidence indicates a few theories contributing to the development of AD, with the leading ones including the amyloid hypothesis, the tau propagation hypothesis, the cholinergic hypothesis and the oxidative stress and inflammatory hypothesis [2,3,4,5]. Amyloid and tau propagation hypotheses relate to direct pathological aspects specific to AD, i.e., β-amyloid (Aβ) protein aggregation and formation of senile plaques outside neurons, and hyperphosphorylation of tau protein and neurofibrillary tangles (NFTs) formation inside neurons [6,7,8]. These theories are also linked with the oxidative stress hypothesis. Oxidative damage in the brains of patients with AD is associated with abnormally marked accumulation of Aβ and deposition of neurofibrillary tangles. Moreover, Aβ does not only induce oxidative stress: its formation also stems from elevated oxidative stress and reactive oxygen species (ROS) generation [2,9,10]. The next concept associated with accelerated pro-aggregation of Aβ is the cholinergic hypothesis, which was originally related to a dysfunction of acetylcholine (ACh)-containing neurons in the brain, substantially leading to the cognitive decline observed in AD. However, several years ago it was reported that two enzymes decomposing ACh, namely acetylcholinesterase (AChE) and butyrylcholinesterase (BuChE), induce aggregation of Aβ fibrils [11]. Since the loss of ACh level contributes to cognitive impairment, primary emphasis has been put on the development of anticholinergic agents that can inhibit both enzymes and increase the levels of ACh in the central nervous system (CNS) [2,11,12].

Basic symptoms of AD, i.e., memory loss and cognitive impairment, are treated with several well-known drugs belonging to a group of acetylcholinesterase inhibitors (AChEIs), which include rivastigmine, donepezil and galantamine. Memantine, being an N-methyl-D-aspartate (NMDA) receptor antagonist, indicated in moderate and severe AD, is an alternative to AChEIs [13,14]. Nevertheless, AD is also accompanied by other behavioral symptoms that are difficult to treat, including aggression, agitation, repetitive vocalizations, wandering, sleep problems, depression and psychosis [15,16,17]. As reported by Ropacki and Jeste [18], psychosis occurs in 41% of AD patients, delusions occur in 36% and hallucinations occur in 18% of the subjects. All these behavioral signs of AD can worsen a cognitive outcome; additionally, their treatment is peculiarly disputable, especially in cases of patients with concomitant diseases [19]. AChEIs and memantine have been found to play an auxiliary role in mild psychosis in individuals with AD; however, they are insufficient for treatment of severe psychotic symptoms [15]. Antipsychotics, especially atypical antipsychotics, appear to be useful therapeutics in alleviating symptoms of psychosis and agitation in AD. The most commonly used antipsychotics are divided into two groups: first-generation antipsychotics (FGA), such as haloperidol, and second-generation antipsychotics (SGAs), such as clozapine, risperidone or olanzapine. This division is based on the difference in the action of drugs representing these two different groups. Atypical antipsychotics have a lower risk of inducting extrapyramidal symptoms, such as muscle stiffness, slowness of movement, tremor and problems with walking, due to the different ways of binding to receptors [20]. Risperidone is an antipsychotic drug officially approved in Europe for behavioral disorders in dementia [19,21]. According to EMA, Risperdal, and its associated names, is an antipsychotic, indicated for the treatment of schizophrenia, manic episodes associated with bipolar disorders and persistent aggression in patients with moderate–severe Alzheimer’s dementia [22]. Other antipsychotics, including quetiapine or olanzapine, are prescribed “off label”. Quetiapine is frequently administered in treatment of neuropsychiatric symptoms in AD, mainly due to its good safety profile and lower incidence of serious side effects, such as extrapyramidal symptoms and tardive dyskinesia, when compared with other antipsychotics [15,23]. In turn, olanzapine appeared to be beneficial in therapy of delusions, hallucinations, anxiety and agitation in AD patients [24]. Although there is some clinical documentation confirming moderate activity of atypical antipsychotics in the management of neuropsychiatric symptoms associated with AD, their effects on cognition are still unexplained and unstructured. Importantly, polypharmacy used in these circumstances imposes an obligation to monitor an increasing risk of adverse side effects of drugs. On the other hand, it enables evaluation of the effects of drugs that can be used “off label”. This approach could have a number of advantages over de novo standard drug development, including shorter times before being introduced to the market and lower costs [25,26,27]. This is of vital importance, especially in view of the recent computational studies of Kumar et al. [2,12,27], which show that some antipsychotic drugs might exhibit encouraging activity against multiple targets associated with AD, including cholinergic neurotransmission, Aβ formation or tau protein deposition.

Our studies evaluate the potential activity of antipsychotic drugs in relation to selected biochemical aspects of AD, which was predicted by Kumar et al. [2] in in silico research. With the application of in vitro research model, we aimed to verify a hypothesis surrounding the anti-AD potential of selected APs. We also attempted to evaluate the relationship between the activities of these compounds and their model structure (derivatives of butyrophenone, diphenylbutylpiperidine, phenothiazine and thiazepine (quetiapine). The aim of this research was to validate the potency of selected antipsychotic drugs on the main hypotheses of AD development. Firstly, we explored in vitro effects of seven antipsychotic drugs (haloperidol, benperidol, bromperidol, promazine, penfluridol, pimozide and quetiapine) on the activity of human AChE and BuChE, and established the kinetic parameters (*K*_m_, *V*_max_) of enzymatic reactions. Furthermore, potential synergism between these antipsychotics and donepezil or rivastigmine towards both cholinesterases (ChEs) was assessed. The next step of this study was to evaluate the impact of antipsychotic drugs on β-amyloid (1–42) (Aβ42) aggregation. Furthermore, the antioxidant potential of antipsychotics has been established using the in vitro human erythrocyte model. Finally, we determined the effects of antipsychotics on the viability of human astrocytes and human umbilical vein endothelial cells (HUVEC) under conditions of induced oxidative stress.

## 2. Results

### 2.1. Cholinesterase Inhibition

**Figure 1:**
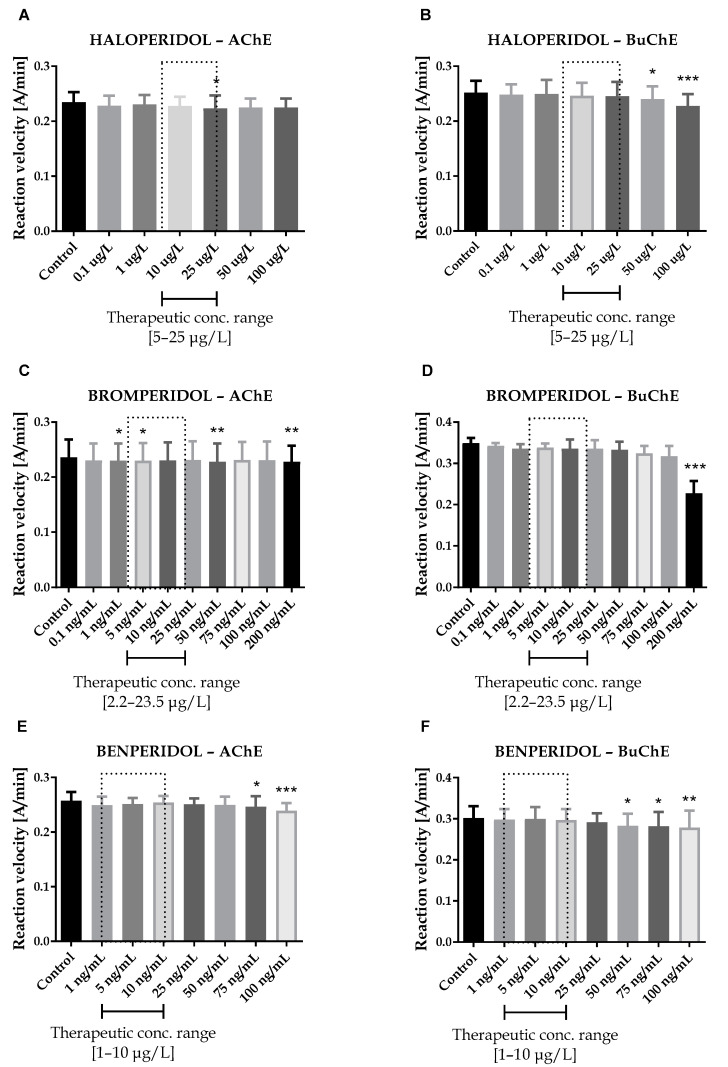
Effects of haloperidol (**A**,**B**), bromperidol (**C**,**D**) and benperidol (**E**,**F**) on AChE and BuChE in vitro activity, respectively. Each data point represents mean ± standard deviation (SD) for at least three independent experiments conducted in duplicates. * *p* < 0.05, ** *p* < 0.01, *** *p* < 0.001 vs. control.

**Figure 2:**
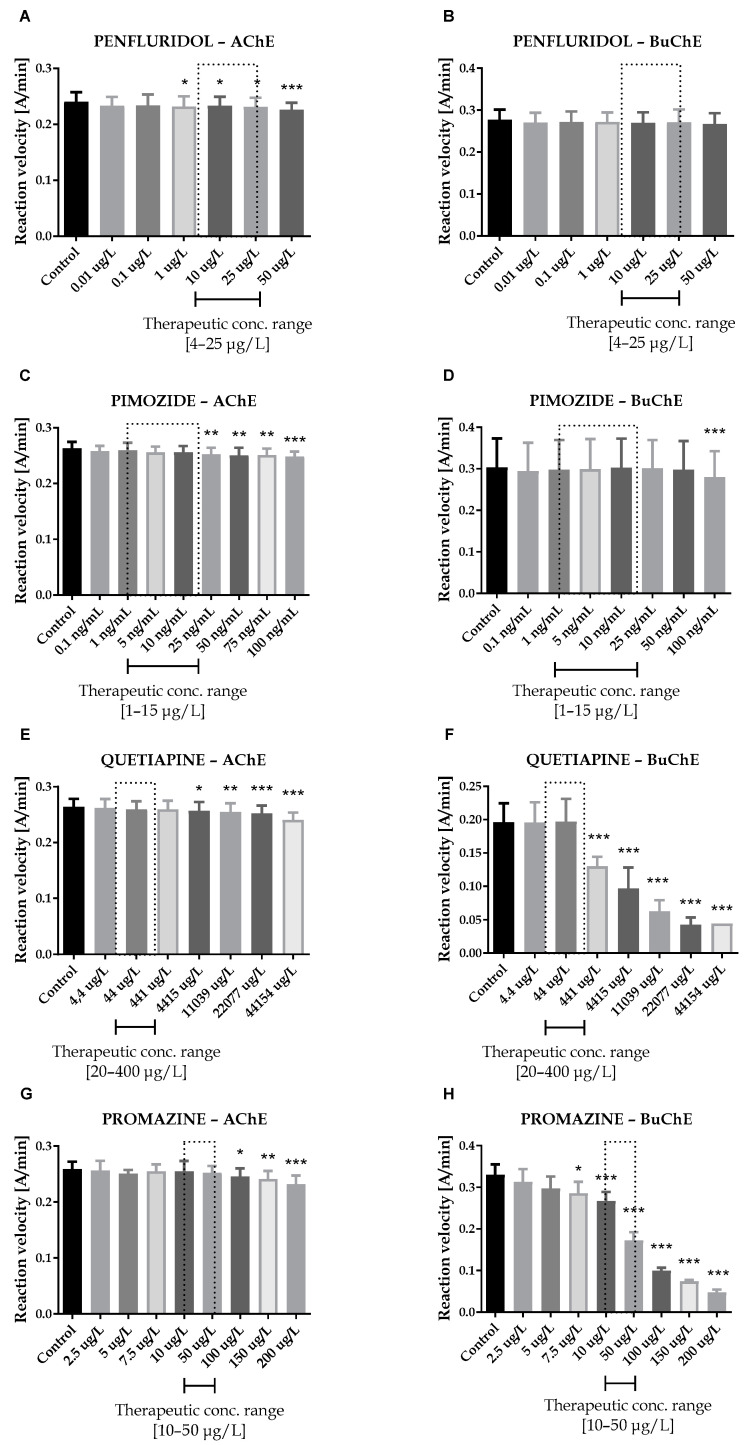
Effects of penfluridol (**A**,**B**), pimozide (**C**,**D**), quetiapine (**E**,**F**) and promazine (**G**,**H**) on AChE and BuChE in vitro activity, respectively. Each data point represents mean ± SD for at least three independent experiments conducted in duplicates. * *p* < 0.05, ** *p* < 0.01, *** *p* < 0.001 vs. control.

Seven antipsychotic drugs, including derivatives of butyrophenone (haloperidol, bromperidol and benperidol), phenothiazine (promazine), diphenylbutylpiperidine (pimozide and penfluridol) and thiazepine (quetiapine), were thoroughly examined in vitro towards the inhibition of human acetyl- and butyrylcholinesterase. Donepezil and rivastigmine, agents which reversibly inactivate ChEs, were used as reference compounds. Donepezil inhibited 50% of AChE and BuChE activity at concentrations of 0.025  ±  0.004 µmol/L and 12.81  ±  1.52 µmol/L, respectively. The corresponding values for rivastigmine were as follows: 64.29 ± 2.97 µmol/L and 0.95 ± 0.09 µmol/L. Effects of antipsychotic drugs on AChE and BuChE reaction velocity are presented in Figure 1A–F and Figure 2A–H.

Therapeutic concentrations of haloperidol do not affect the activity of esterases. At higher concentrations (50–100 µg/L), it significantly reduces the activity of BuChE. For instance, haloperidol at 100 µg/L decreased BuChE activity by 9.6 ± 2.3% (0.228 ± 0.021 A/min vs. 0.252 ± 0.022 A/min for control). Other butyrophenone derivatives, bromperidol and benperidol, significantly decreased AChE and BuChE activity at concentrations higher than therapeutic plasma concentrations (TPC). Bromperidol significantly diminished the activity of both ChE at 200 µg/L (0.228 ± 0.029 A/min vs. 0.236 ± 0.032 A/min for control in the case of AChE and 0.230 ± 0.023 A/min vs. 0.349 ± 0.013 A/min for control in the case of BuChE), while benperidol exerted comparable anti-ChE effects at 75–100 µg/L (0.250 ± 0.015 A/min–0.239 ± 0.014 A/min vs. 0.258 ± 0.016 A/min for AChE control and 0.283 ± 0.029 A/min–0.279 ± 0.021 A/min vs. 0.302 ± 0.029 A/min for BuChE control). Compounds at concentrations higher than TPC—penfluridol at 50 µg/L (0.226 ± 0.012 A/min vs. 0.241 ± 0.017 A/min for control), pimozide 25–100 µg/L (0.253 ± 0.012 A/min–0.248 ± 0.009 A/min vs. 0.263 ± 0.011 A/min for control), quetiapine over 4000 µg/L (0.257 ± 0.016 A/min–0.241 ± 0.013 A/min vs. 0.264 ± 0.014 A/min for control) and promazine at 100–200 µg/L (0.245 ± 0.015 A/min–0.232 ± 0.016 A/min vs. 0.259 ± 0.013 A/min for control)—significantly inhibited AChE activity. The most profound effects on the activity of BuChE were reported in the case of promazine and quetiapine, which allowed for IC_50_ values calculations (Table 1). These results can be associated with the presence of a tricyclic system containing nitrogen and sulphur in quetiapine and promazine structure.

### 2.2. Kinetic Parameters of Enzymatic Reaction Estimation

**Figure 3:**
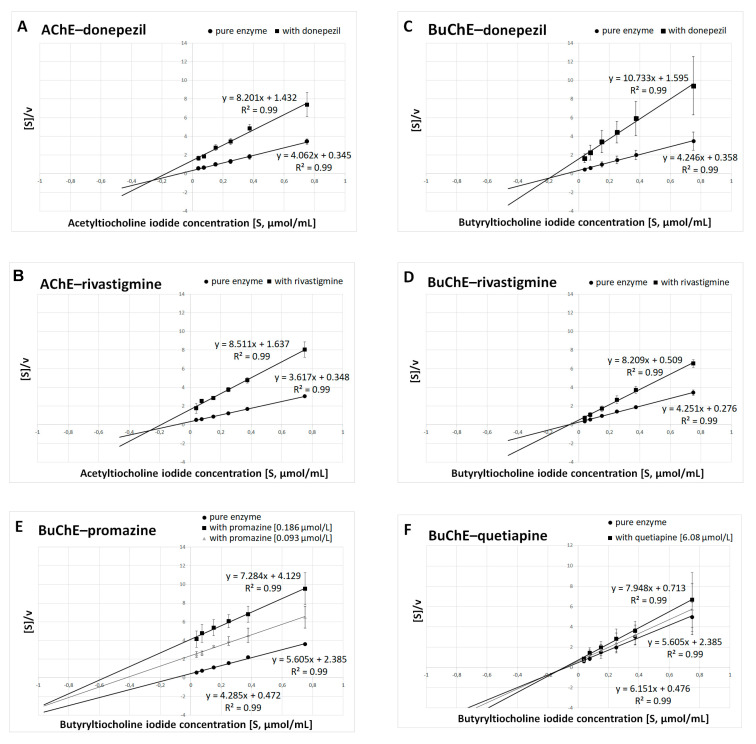
Determination of kinetic parameters of enzymatic reactions. The Hanes–Woolf plots were used to calculate the maximal velocity (*V*_max_) and the Michaelis–Menten constant (*K*_m_). (**A**) AChE and donepezil at a concentration of 0.025 μmol/L. (**B**) AChE and rivastigmine at a concentration of 64.29 μmol/L. (**C**) BuChE and donepezil at a concentration of 12.81 μmol/L. (**D**) BuChE and rivastigmine at a concentration of 0.95 μmol/L. (**E**) BuChE and promazine at a concentration of IC_50_ = 0.186 μmol/L and ½ IC_50_ = 0.093 μmol/L. (**F**) BuChE and quetiapine at a concentration of IC_50_ = 6.08 μmol/L and ½ IC_50_ = 3.04 μmol/L. Results are presented as mean ± SD for two or three independent experiments, conducted in duplicates on various erythrocytes for AChE and plasma for BuChE.

Kinetic parameters were obtained on the basis of three individual experiments carried out on different biological materials. For this purpose, various concentrations of substrates (ATC and BTC) were used in experiments. The type of inhibition and kinetic parameters of enzymatic reactions were obtained by linear regression using the Hanes–Woolf equations. The *K*_m_ and *V*_max_ values obtained for the pure enzyme and *K*_m(i)_ and *V*_max(i)_ for the tested compounds at IC_50_—and additionally ½ IC_50_ for promazine and quetiapine concentrations—were used to determine the type of inhibition (Figure 3A–F).

Summarized results of *K*_m_ and *V*_max_ are presented in Table 2A for donepezil, rivastigmine, promazine and quetiapine at concentrations equal to their IC_50_ values; and in Table 2B for promazine and quetiapine at concentrations equal to their ½ IC_50_ values. Donepezil inhibits AChE and BuChE in a mixed manner, which is in accordance with previous findings [34,35,36,37]. It was established that rivastigmine inhibited BuChE noncompetitively, as *K*_m(i)_ remained constant in comparison with *K*_m_, while *V*_max(i)_ decreased. In the case of AChE, rivastigmine exhibited mixed inhibition (*K*_m(i)_ increased, whereas *V*_max(i)_ decreased). The same type of inhibition revealed promazine and quetiapine in relation to BuChE.

### 2.3. Potential Synergism between Antipsychotics and AChEIs towards Inhibition of Human ChE

Synergism studies assessed the effect of antipsychotic drugs on anti-ChE properties of known ChEIs. Within this study, we performed an assay that used binary mixtures to investigate the influence of the tested antipsychotics on the donepezil and rivastigmine IC_50_ values. For this purpose, donepezil was studied at 0.1–100 nmol/L for AChE and 0.2–100 μmol/L for BuChE, whereas rivastigmine was investigated at concentrations of 5–100 μmol/L and 0.05–5 μmol/L, regarding AChE and BuChE, respectively. As presented in Table 3 and Table 4, tested antipsychotic drugs in most cases increase the anti-BuChE potential of donepezil and the anti-AChE properties of rivastigmine. Penfluridol, which does not affect the anti-AChE activity of rivastigmine, is an exception. A combination of donepezil and promazine, benperidol, bromperidol or quetiapine at TPC_max_ demonstrates the highest anti-BuChE activity. Mixtures of donepezil, containing the mentioned antipsychotics lowered the IC_50_ value by 51.8–64.8% in comparison to pure donepezil. The greatest effect regarding AChE activity was found for a combination of rivastigmine, pimozide, bromperidol, quetiapine and benperidol at their TPC_max_. The IC_50_ value of binary mixtures of rivastigmine with these substances were 38.2–51.0% lower than the IC_50_ value of pure rivastigmine. As it follows from the above, the butyrophenone-structured compounds with a fluorine atom attached to the aromatic ring show synergistic activity with both donepezil and rivastigmine, enhancing their anti-BuChE and anti-AChE effects, respectively. Phenothiazine and thiazepine derivatives (promazine and quetiapine, respectively), which contain nitrogen and sulphur in a tricyclic system, exhibit the same properties. Among the tested phenylpiperidine derivatives, such properties are exhibited by pimozide, which benzimidazole substituents, in contrast to penfluridol, with a benzene ring with attached halogen atoms.

**Figure 4:**
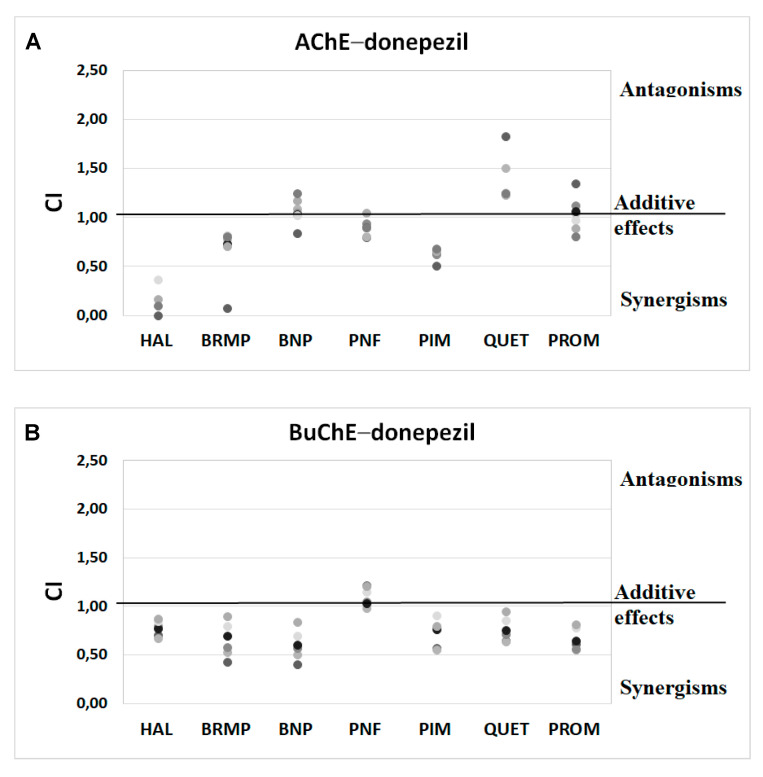
Analysis of potential synergism between donepezil (DON) and haloperidol (HAL), bromperidol (BRMP), benperidol (BNP), penfluridol (PNF), pimozide (PIM), quetiapine (QUET) and promazine (PROM) with the application of the median–effect principle. Data from an AChE (**A**) and BuChE (**B**) inhibitory activities assay were analyzed with the Chou–Talalay method. Results are presented as CI values determined with ComboSyn software for binary mixtures with variable concentration of donepezil (range 0.005–0.1 µmol/L for AChE and 2–100 for BuChE) and constant concentration of antipsychotic (at their TPC_max_). The darker marker means higher concentration of DON in binary mix. The IC values for an individual concentration of DON are shown in Table S1 (Supplementary Materials). CI—combination index: where CI < 1, =1 and >1 indicate synergism, additive effect and antagonism, respectively.

**Figure 5:**
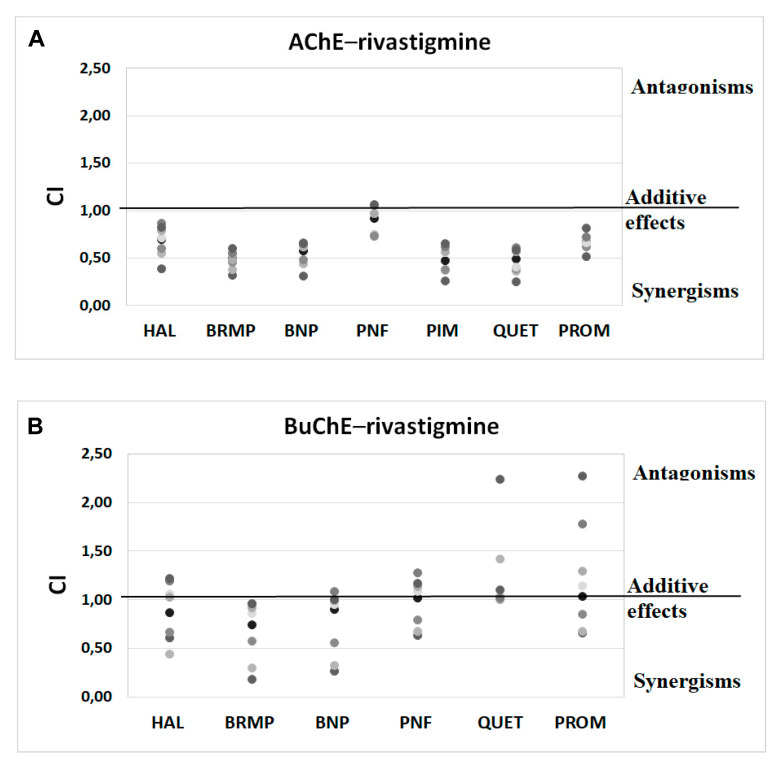
Analysis of potential synergism between rivastigmine (RIV) and haloperidol (HAL), bromperidol (BRMP), benperidol (BNP), penfluridol (PNF), pimozide (PIM), quetiapine (QUET) and promazine (PROM) with the application of the median–effect principle. Data from an AChE (**A**) and BuChE (**B**) inhibitory activities assay were analyzed with the Chou–Talalay method. Results are presented as CI values determined with ComboSyn software for binary mixtures with variable concentration of rivastigmine (range 5–100 µmol/L for AChE and 0.05–5 for BuChE) and constant concentration of antipsychotic (at their TPC_max_). The darker marker means higher concentration of RIV in binary mix. The IC values for an individual concentration of RIV are shown in Table S2 (Supplementary Materials). CI—combination index: where CI < 1, =1 and >1 indicate synergism, additive effect and antagonism, respectively. Figure 5B does not show a relationship between the interaction of rivastigmine/pimozide on BuChE, which ComboSyn calculated to be above 2.5, beyond the scale of the plot.

Anti-ChE results of drug combinations were also verified by ComboSyn software [38]. As presented in Figure 4 and Figure 5, the Chou–Talalay analysis [38] confirms our experimental results, which have proved to increase the anti-BuChE effects of donepezil, and the anti-AChE effects of rivastigmine. Additionally, the Chou–Talalay analysis indicates a dose-dependent effect of antipsychotic drugs. For instance, an antagonistic effect is observed for lower concentrations of the donepezil–promazine mixture in the case of anti-AChE activity, while in higher doses, this effect is reduced and synergism can even be observed. The rivastigmine–promazine mixture, which changes from synergistic to antagonistic with its increasing concentration, demonstrates the opposite outcome—BuChE inhibition.

The values of CI of binary mixtures donepezil and rivastigmine with tested antipsychotics are included in the Supplementary Materials (Tables S1 and S2).

### 2.4. Beta-Amyloid Aggregation Studies

**Figure 6:**
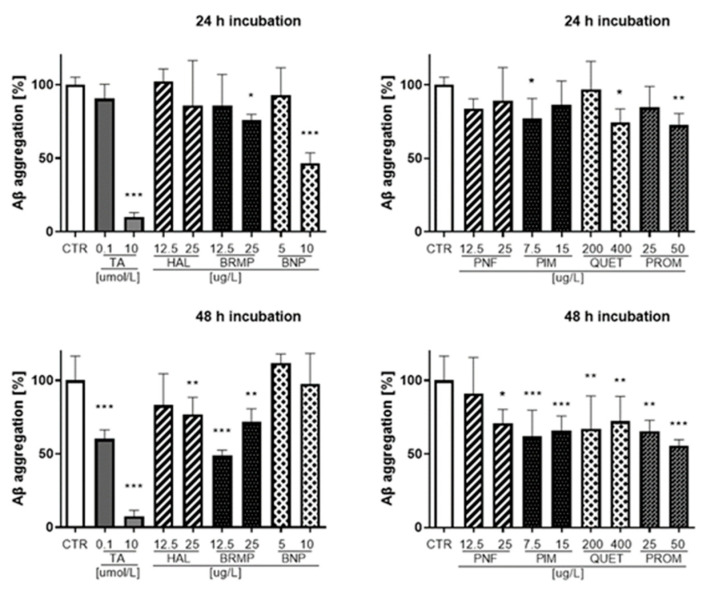
Influence of antipsychotics at the TPC and ½ × TPC on Aβ aggregation measured after 24 and 48 h. Tannic acid at a concentration of 0.1 μmol/L and 10 μmol/L (170.12 μg/L and 17,012 μg/L, respectively) was used as an inhibitor of Aβ aggregation. Mean ± SD, *n* = 3–9. * *p* < 0.05, ** *p* < 0.01, *** *p* < 0.001, vs. control which constituted 100% Aβ aggregation.

The effects of antipsychotics on the aggregation of Aβ were examined using fluorescent properties of Thioflavin T (ThT) dye. Fluorescence measurements were performed for antipsychotics at the TPC and ½ × TPC. The obtained results, presented as a percentage of Aβ aggregation, are included in Supplementary Materials for 10, 30, 60 and 90 min incubation (Figures S1 and S2, Supplementary Materials), whereas those for 24 and 48 h are shown in Figure 6.

The strongest inhibition of Aβ (42.1–53.3%) was observed for benperidol at a concentration of 10 μg/L in the time range from 10 min to 24 h incubation. Benperidol revealed the lowest anti-Aβ aggregation properties after 48 h even at the highest concentration (10 μg/L). At the last time point of the fluorescence measurement (48 h), the highest percentage value of Aβ inhibition was reported for bromperidol (12.5 μg/L) and represented 51.2% compared with the control (untreated samples), which constituted 100% of Aβ aggregation.

### 2.5. ROS-Induced RBCs Hemolysis

Potential antioxidant properties of antipsychotic drugs were studied, i.e., their ability to protect erythrocytes from oxidative damage induced by 2,2′-azobis(2-methylpropionamidine) dihydrochloride (AAPH), as well as the formation of methemoglobin—another marker of erythrocyte oxidative stress. Preliminary experiments enabled us to establish the appropriate concentration of AAPH in these studies, i.e., 50 mmol/L.

Results of the studies evaluating the effects of the compounds alone on erythrocyte hemolysis and their methemoglobinogenic properties are included in the Supplementary Materials (Figures S3 and S4). The influence of antipsychotic drugs on RBC hemolysis did not exceed 5%, which indicates biocompatibility of these compounds. The only exception among the compounds tested was promazine, which at 250 μg/L increased disintegration of the erythrocyte membrane by over 45% and 59% after 5 and 24 h of incubation, respectively (Figure S3, Supplementary Materials). Promazine at 250 μg/L also contributes to increased formation of methemoglobin; after 5 h of incubation, it was 39% and after 24 h it was 64% (Figure S4, Supplementary Materials).

**Figure 7:**
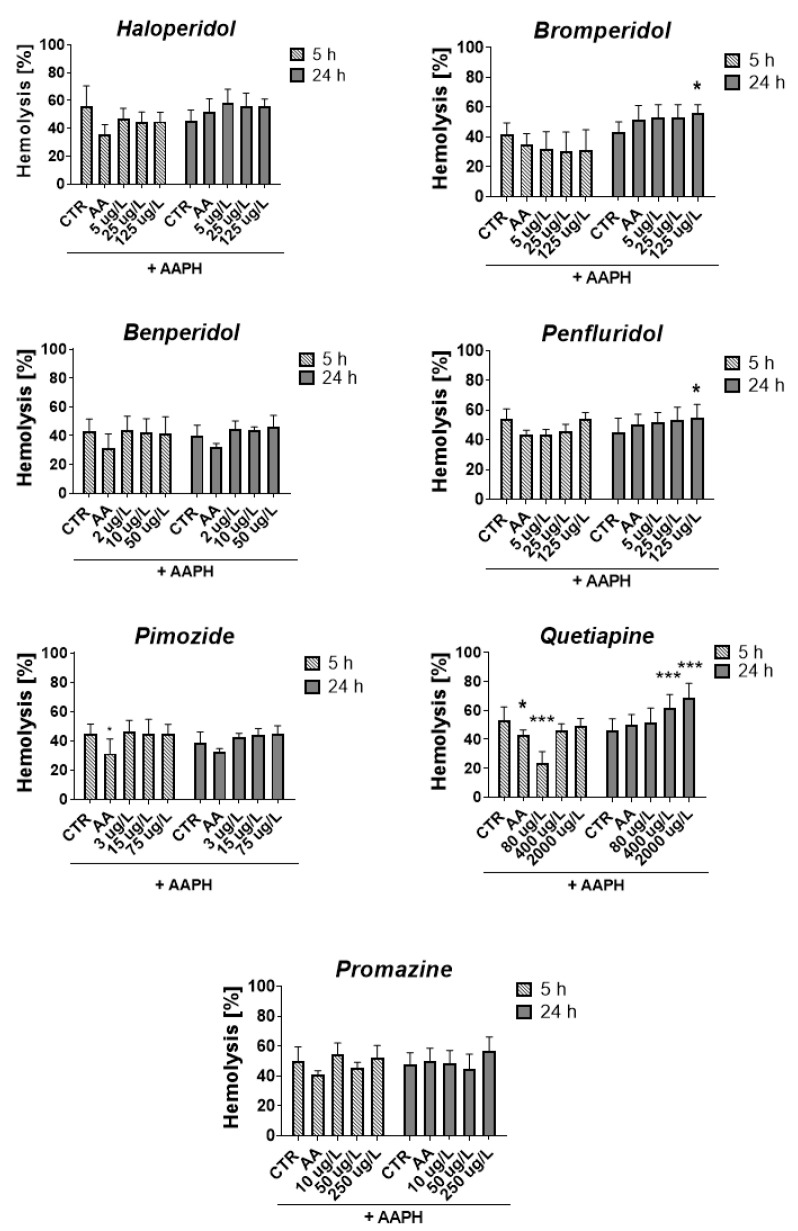
Effect of tested antipsychotics at 1/5 × TPC_max_, TPC_max_ and 5 × TPC_max_ on erythrocyte hemolysis measured after 5 h and 24 h of incubation at 37 °C. Results are presented as mean ± SD, *n* = 4–12. * *p*  <  0.05, *** *p* < 0.001 vs. control (samples with AAPH).

Figure 7 shows the percentage of RBC hemolysis measured after 5 and 24 h incubation of erythrocytes with compounds and AAPH.

After 5 h, all compounds slightly reduced or did not contribute to the lysis of RBCs, except for quetiapine at a concentration of 80 μg/L, which produced a statistically significant decrease in RBCs hemolysis compared with the control with AAPH (23.9% vs. 53.4% for AAPH control, *p* < 0.001). The opposite effect was produced after 24 h of incubation, in which enhanced hemolysis was observed (51.9% vs. 45.9% for AAPH control, *p* > 0.05). It may indicate a dependence of the hemolytic effect on the time of exposure to quetiapine since, in the case of all tested concentrations of quetiapine, an increase in the hemolytic effect was reported over time between 5 and 24 h. Similar dependence was reported for bromperidol, for which hemolysis increased over time for all tested concentrations.

Regarding the methemoglobin formation, the statistically significant impact was reported only for quetiapine and promazine, for which a graphical presentation of the results is included in the Supplementary Materials (Figure S5, Supplementary Materials). The 5 h incubation with quetiapine at 80 and 400 μg/L resulted in a significant decrease in methemoglobin formation compared with the control with AAPH (17.1% and 18.8% vs. 61.9% for AAPH control, *** *p* < 0.001, respectively). In the case of promazine at 10 and 50 μg/L decrease in heme oxidation in erythrocytes was observed after 24 h of incubation (60.7% and 54.5% vs. 78.1% for AAPH control, *** *p* < 0.001, respectively).

### 2.6. Antioxidative Potential of Antipsychotics in Cell Culture Model

In order to further characterize the potential antioxidant properties of the tested compounds, we conducted experiments with human umbilical vein endothelial cells (HUVEC) and human astrocyte cells. These types of cells were chosen because metabolic processes in some types of brain cells, including neurons, and endothelial cells, while largely different and variable, are complementary to assure proper functioning of the brain [39]. The WST-1 test was used to assess the effect of antipsychotic drugs (under physiological conditions and AAPH-induced oxidative stress) on HUVEC and astrocyte cells viability. First, cells were stimulated with tested compounds at concentrations corresponding to ½ × TPC and TPC for 24 h. Incubation with compounds at all tested concentrations did not significantly affect any of the cell lines (HUVEC and human astrocytes) viability (Figure S6, Supplementary Materials).

**Figure 8:**
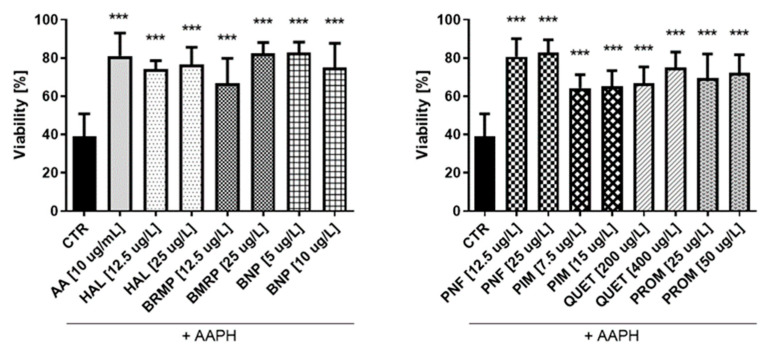
Potential antioxidative effect of selected antipsychotics in HUVEC cells. The experiments were performed by assessing the viability of HUVEC cells using the WST-1 assay. The cells were incubated for 1 h with the test compounds, and then AAPH, which was an inducer of oxidative stress, was added. The results are presented relative to control treated with pure medium (100% viability). An asterisk denotes a statistically significant difference between the control (CTR, in which were cells treated with pure AAPH [17.5 mmol/L]) and samples co-treated with antipsychotics and AAPH; *** *p* < 0.001.

In the next step of the studies, the antioxidant potential of antipsychotic drugs was investigated. The effects of AAPH, an oxidizing agent, at the concentration of 17.5 mmol/L on the viability of HUVECs and 15 mmol/L for astrocyte were also determined. Samples treated with ascorbic acid (AA) at a concentration of 10 µg/mL were performed as well. In the case of HUVEC cells (Figure 8), the tested antipsychotics showed protective effect on cells against oxidative stress at level comparable to AA (10 μg/mL), which corresponded to 81.0 ± 3.4%. Results of the conducted research indicate antioxidative properties of tested antipsychotics in these cells. The tested compounds significantly increased the viability of HUVEC to 64.2–82.9% (for ½ × TPC pimozide and ½ × TPC benperidol, respectively) as compared with control samples with AAPH (17.5 mmol/L), which decreased cells viability by approximately 60.0%. Bromperidol and quetiapine showed a potential relationship between the dose and the antioxidant effect. For bromperidol, these values were 66.8% and 82.5% (for concentrations 12.5 μg/L and 25 μg/L, respectively), while for quetiapine they were 66.8% and 74.9% (200 μg/L and 400 μg/L, respectively). An analysis of the effectiveness of compounds at both concentrations: ½ × TPC and TPC, revealed they the most pronounced protective effect on viability of HUVEC, corresponding to 80.5% and 82.9%, was observed for penfluridol, whereas cells incubated with pimozide demonstrated the lowest viability, i.e., 64.2% and 65.2%.

**Figure 9:**
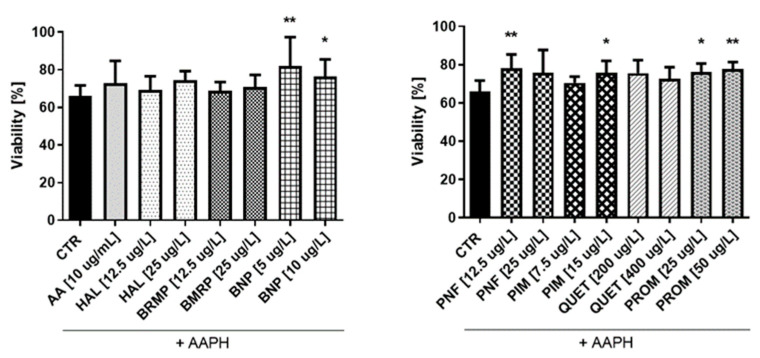
Potential antioxidative properties of tested antipsychotic drugs in astrocytes. The measurements of cell viability were assessed using a WST-1 assay. The cells were stimulated for 1 h with the tested compounds, and later AAPH, an oxidative stress inducer, was added. The results are presented relative to control treated with pure medium (100% viability). Calculations show a statistically significant difference between the control (CTR, comprising cells treated with pure AAPH [15 mmol/L]) and samples co-treated with antipsychotics and AAPH; * *p*  <  0.05, ** *p* < 0.01 vs. control (samples with AAPH).

In the case of astrocytes (Figure 9), it was also found that some tested compounds protect cells against oxidative stress at a level comparable to AA (10 μg/mL), for which viability was 72.9%. Only four substances—benperidol (5 and 10 μg/L), penfluridol (12.5 μg/L), pimozide (15 μg/L) and promazine (25 and 50 μg/L)—showed a statistically significant increase in the astrocyte viability compared with the control, where cells treated with pure AAPH were used. The greatest impact (81.9%) was revealed in the case of benperidol at a concentration of 5 μg/L, whereas the lowest one (68.9%) was revealed in the case of bromperidol at a concentration of 12.5 μg/L.

## 3. Discussion

Symptoms of AD do not only include memory loss and cognitive decline, but also include neuropsychiatric manifestations, such as agitation and aggressiveness. These AD-related symptoms are usually treated with antipsychotics; however, their effects on cognition and safety in AD remain unexplored [15]. Nevertheless, antipsychotic drugs (especially those belonging to the group of atypical antipsychotics) are often used as drugs of choice in treatment of behavioral and psychiatric symptoms in people with AD [15,16,17,24]. According to da Silva et al. [40], randomized, placebo-controlled, multicenter trials showed that antipsychotics do not appear to improve patients’ condition, so their care needs are still the same, but the lack of safer alternatives enforces the use of antipsychotic drugs for neuropsychiatric symptoms of dementia [40,41,42]. For this reason, it is essential to investigate not only the anti-AChE and anti-BuChE effects of antipsychotic drugs, but also their potential synergistic or antagonistic effects with drugs used in the treatment of AD.

This work aimed to determine the potency of selected antipsychotic drugs in in vitro studies against the main hypotheses associated with AD development that could be reflected in clinical application through drug repurposing. The process of developing new applications of a drug beyond its original use or commercially approved indication [43] is a quite new idea, known as drug repurposing or reprofiling. It is believed that drug repurposing offers greater benefits over de novo drug discovery [44]. Repurposing also allows faster identification of new therapies for diseases, particularly in those cases where preclinical safety studies have already been accomplished [37]. Kumar et al. [2] adopted a computational method based on the ligand–protein interaction in order to explore potential antipsychotic drugs for treatment of AD. The authors found that some antipsychotic drugs might exhibit encouraging potential against multiple targets associated with AD. In this study, Kumar performed molecular docking for approximately 150 antipsychotic drugs and the best drugs were identified on the basis of dock score and glide energy [2]. The top hits were then compared with the already known inhibitor of the respective proteins. Some of the antipsychotic drugs, including benperidol, bromperidol, pimozide and promazine hydrochloride, showed relatively better docking score and binding energies as compared with the already known inhibitors of the respective targets. However, with the in silico repurposing approach, there might be a possibility of false-positive hits during screening and also the activity of the candidate drug molecules may vary in the in vitro or in vivo systems. Nevertheless, the search for the potential of antipsychotic drugs as anti-AD drugs is justified because they all cross the blood–brain barrier (BBB). Therefore, there is a chance that they will also act on the main points of the hypothesis of the pathomechanism of AD in vivo. Accordingly, we decided to validate the potency of selected antipsychotic drugs in in vitro studies on three hypotheses of AD development, i.e., cholinergic hypothesis, β-amyloid and oxidative stress. For this purpose, we used various research models using biological material and cell culture methods.

Regarding ChEs inhibition, the most profound effects were reported for promazine and quetiapine, which allow the calculation of BuChE IC_50_ values. Calculation of the SI confirmed that both of these compounds are characterized by greater selectivity towards BuChE than towards AChE. Comparing the obtained IC_50_ values with the values of clinically registered drugs, it was found that promazine shows higher anti-BuChE activity relative to donepezil and rivastigmine, as does quetiapine, but only in relation to donepezil. These results are beneficial in view of the studies conducted by Grossberg et al. [45], who reported that the BuChE activity in cholinergic neurotransmission increases in AD by 40–90%; although, BuChE makes up only 10% of the total activity of ChE in the cortex of healthy human brain. Importantly, selective BuChE inhibition could be clinically valuable due to an improvement of cognitive function [45,46]. These results are in line with the recent outcomes which highlight the fact that rivastigmine [47,48] improves cognitive functions in AD patients by centrally inhibiting not only AChE but also BuChE. Similarly, our research confirmed the reports on the inhibition of BuChE by promazine. Debord et al. [49] found that phenothiazine derivatives (i.e., promazine) present the ability to inhibit ChEs, with specificity for BuChE. Structure–activity relationships, related to the binding of phenothiazines to BuChE, were developed. In this study, the authors used the phenothiazine derivative with the N-diethylamino group ethopropazine as a model compound in the docking program to create a molecular model of formation of the complex with BuChE. These molecular docking studies showed the active site of this enzyme: the phenothiazine ring interacts with a residue of tryptophan, whereas the peripheral chain interacts with a phenylalanine residue in the BuChE structure [49,50].

The next step in our research was to evaluate the synergism of antipsychotic drugs with known AChEIs. The obtained results confirm promazine and quetiapine have a potential clinical value in treatment of AD due to the fact that they enhance the BuChE inhibition by donepezil by over 50%. Within this study, it was also found that other tested antipsychotic drugs increase the anti-BuChE properties of donepezil and the anti-AChE properties of rivastigmine, except for penfluridol, which did not affect the AChE IC_50_ value for rivastigmine. According to the clinical point of view, these results should be taken into account in determining the dosage of drugs in treatment of patients with AD and comorbidities. A combination therapy of AD patients may be preferred due to its efficacy and the ability to reduce the dosage of donepezil or rivastigmine, if necessary. This, in turn, may result in mitigated side effects, which are often caused by the use of donepezil or rivastigmine.

A crucial feature in AD are amyloid plaques, which are composed of aggregated Aβ fibrils. The hypothesis that Aβ plays a key role in AD pathogenesis was proposed by Hardy in 1991 [51,52], and till now, inhibition of Aβ aggregation is of scientific interest to many researchers. Many pharmaceutical companies have developed new agents that are at different stages of clinical trials targeting the amyloid cascade [53,54,55]. These advanced studies resulted in a development of the first new drug for the treatment of Alzheimer’s disease in two decades. On 7 June 2021, the US Food and Drug Administration (FDA) approved aducanumab [56], a human monoclonal antibody selective for aggregated forms of Aβ [57]. Results of clinical studies support the observation that an aducanumab therapy reduces brain Aβ plaques and that reduction in Aβ plaques provides a clinical benefit for AD patients [58]. Aβ fibrils formation can be divided into the following phases: the nucleation phase, the elongation phase and the stationary phase [59,60]. Monomers attach to each other to form larger complexes, ranging from dimers to heptamers. They next grow into larger oligomers that eventually form protofibrils, from which mature fibrils are formed [61,62,63]. Literature reports show that the process of deposit formation (fibrils) proceeds through the stage of oligomers, characterized by a much higher toxicity than monomeric forms. Presence of oligomers contributes the most to the degeneration of synapses and to the occurrence of disease symptoms [64,65]. Taking into account the multistage nature of Aβ aggregation, it can be concluded that the inhibition of the early phase of aggregation inhibits the formation of smaller Aβ aggregates, while tests carried out at several-hour intervals indicate inhibition of the formation of large fibrils. Results of our research indicate that most of the tested antipsychotics at concentrations of ½ TPC and TPC significantly reduces Aβ aggregation within 10–90 min, except for penfluridol at a concentration of 25 µg/L, which does not inhibit Aβ aggregation at any of these time points. It can also be noticed that within 90 min of incubation, haloperidol (at both tested concentrations) also ceases to significantly inhibit Aβ aggregation. Moreover, our results obtained after 24 and 48 h may also be potentially clinically relevant. In this case, bromperidol, pimozide, quetiapine and promazine (all at both tested concentrations) are essential. A significant decrease in Aβ aggregation after longer time of incubation, probably associated with fibrils generation, was noted. Presented results could become a starting point for further research that would contribute to a development of a drug which could prevent Aβ aggregation, due to the fact that only in silico data are available in this field [2,11,27]. However, it should be noted that the conditions of the tests carried out relate to in vitro conditions, which generates the need to conduct further in vivo tests.

Oxidative stress is scientifically described as imbalance between the generation of reactive oxygen or nitrogen species (ROS/RNS) and the cell capacity to counterbalance them by antioxidant cellular mechanisms [66]. An impressive amount of evidence supports the hypothesis that oxidative stress is an early and substantial pathogenic factor in AD. For instance, a study of Bradley et al. [67] found increased brain levels of 4-hydroxyhexenal (HHE), a marker of lipid peroxidation, in early stages of AD. Similar results have also been reported for other α, β-unsaturated aldehydes, such as 4-hydroxynonenal (HNE) and acrolein, in vulnerable regions of mild cognitive impairment, preclinical AD, and late-stage AD brains [68]. These aldehydes are highly reactive and can easily modify proteins [65]. Additionally, markers of protein oxidation, such as protein carbonyls, have been found to increase in AD brains in areas with histopathological AD features [69]. Oxidative stress is not only a pathological hallmark of AD, but it is also regarded as a factor involved in the initiation of the disease. In fact, oxidative stress and concomitant cellular damage were found to be the first noticeable features in AD progression [70]. Importantly, there is a clear relationship between β-amyloid and oxidative stress. It has also been established that β-amyloid contributes to extensive ROS production, leading to mitochondrial damage [66]. Aβ-binding alcohol dehydrogenase (ABAD) in mitochondria may be a link between β-amyloid and oxidative stress. The interaction between β-amyloid and ABAD was found to increase ROS formation, mitochondrial dysfunction, and finally apoptosis [71]. Apart from elevated markers of oxidative stress, there is also evidence for decreased antioxidant power in the brain. All these factors prompted scientists to search for alternative methods of treating AD, with particular emphasis put on compounds with antioxidant activity [72].

Determination of antioxidative potential is also important in view of the fact that AD is often associated with comorbidities [73]. According to the old data [74,75], treatment with antipsychotics might be associated with oxidative stress, which has been regarded as one of mechanisms in the pathogenesis of extrapyramidal side effects. However, more recent outcomes suggest that only typical neuroleptics are associated with the risk of oxidative damage, unlike atypical drugs such as olanzapine or aripiprazole [76]. Having taken the aforementioned arguments into account, we decided to conduct comprehensive studies aiming to evaluate the antioxidant potential of several antipsychotics. These studies were performed with the use of two different models. The first type of study was conducted using human red blood cells. Despite the fact that erythrocytes, due to the lack of nucleus, do not make for a typical cell model, they were selected for this study based on their function in oxygen and carbon dioxide transport and high heme (Fe) content. In addition, erythrocytes are fragile and highly susceptible to cell membrane damage, which can lead to hemolysis. Therefore, hemolysis constitutes a very good model for studying free-radical-induced oxidative stress and for assessing the antioxidant activity of xenobiotics [77]. In this study, we determined the antioxidative potential of commonly administered antipsychotics in erythrocytes. The compounds were incubated with 2% RBC suspension for 5 and 24 h followed by determination of the hemolysis rate. None of the tested compounds except for promazine at 250 µg/L, which is 5-fold higher than TPC, contributed to a substantial increase in RBC hemolysis (data available on request). On the basis of these promising results, we conducted subsequent studies using AAPH, which is a well-known oxidizing agent. Most of studied antipsychotics did not affect AAPH-induced erythrocyte hemolysis. The most intriguing effects were reported for quetiapine at a concertation of 80 μg/L, for which a significant decrease (*p* < 0.001) in the hemolysis rate was noted after 5 h incubation. On the contrary, this effect was ameliorated after 24 h incubation, while for higher concentrations of quetiapine (400 and 2000 µg/L), an even greater percentage of hemolyzed erythrocytes was observed. We suppose that this result is associated with the exhaustion of the defense antioxidative mechanisms in erythrocytes after 24 h of incubation. The observed moderate antioxidative effect of quetiapine corresponds to the results reported by Lian et al. [78], who found that co-administration of quetiapine exerts protective effects on the catalase and total superoxide dismutase, and blocks ethanol-induced oxidative stress. In the research of Sadowska-Bartosz et al. [79], a comparison was made between the antioxidant activities of six atypical antipsychotic drugs—clozapine, quetiapine, olanzapine, risperidone, ziprasidone and aripiprazole—as well as a typical antipsychotic drug, haloperidol. Authors used several tests of antioxidant activity evaluation performed in vitro under conditions of generated oxidative stress. In most of the tests, olanzapine showed the highest antioxidant activity, followed by clozapine, with the other compounds being much less active or not active at all. Clozapine and olanzapine, similarly to quetiapine, are nitrogen-containing molecules and behave as Lewis bases donating electrons. In these drugs, the nitrogen is linked to an alkyl group, which facilitates electron donation and hydrogen donation from the amino group. The radical produced can resonate with the aromatic ring, which stabilizes its structure.

**Figure 10:**
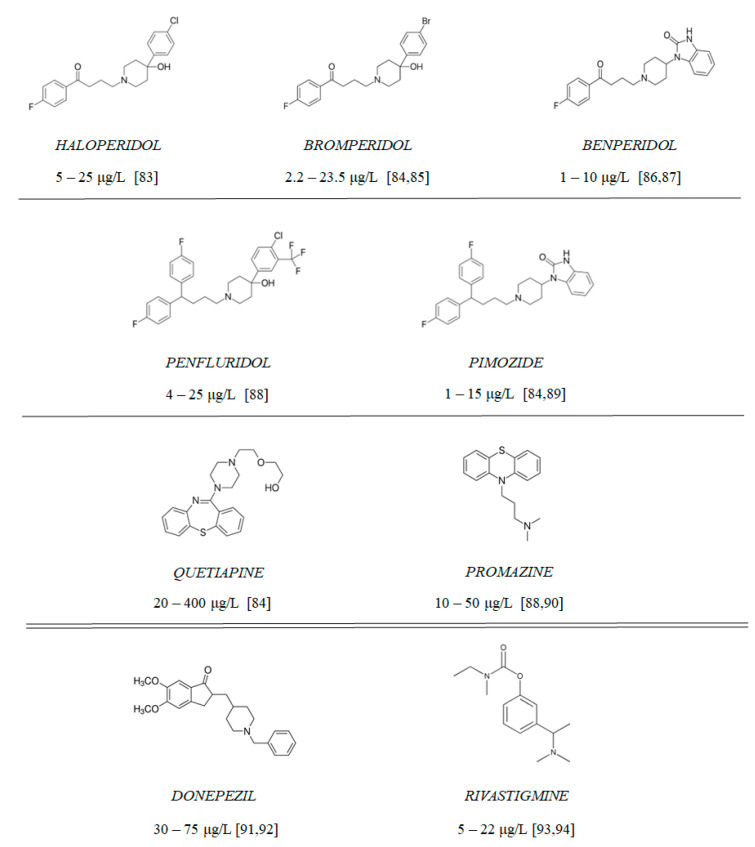
Chemical structure of tested antipsychotics (with therapeutic plasma concentrations according to the literature [83,84,85,86,87,88,89,90,91,92,93,94]) and clinically approved AChE inhibitors: rivastigmine and donepezil.

The second part of the research was performed on a cell model using human endothelial cells and astrocytes. These types of cells were chosen because metabolic processes in some types of brain cells, including neurons, and endothelial cells, although largely different and variable, are complementary to assure proper functioning of the brain [39,80]. Similar to the RBC model, the first step of the studies included estimation of the effects of pure antipsychotics on the viability of HUVECs and astrocytes. All the tested compounds at their plasma therapeutic concentrations were found not to affect significantly the viability of any of the cell lines. Due to the fact that these are the first studies of this type, we cannot compare our results with other studies. The publication of Wiklund et al. [81] is an exception, where the authors did not report any significant effect of haloperidol on the viability of HUVEC. The last stage of the study included an assessment of HUVEC and astrocyte viability upon co-treatment with antipsychotics and AAPH (Figure 9 and Figure 10). It was showed that in HUVEC, all compounds tested at both concentrations significantly increased cell viability compared with the control (samples treated with pure AAPH). In contrast, with regard to astrocytes, only benperidol and promazine showed antioxidant potential at both tested concentrations. Surprisingly, astrocytes were found to be less sensitive to the antioxidant effects of antipsychotic drugs compared with HUVEC.

These results are very important because astrocytes are considered the most sensitive sensors, regulators and protectants of neural functions [82].

## 4. Materials and Methods

### 4.1. Materials

#### 4.1.1. Tested Compounds

Compounds 1–9 (Figure 10) include 7 antipsychotic drugs which exhibit different chemical structures and have different leading structures: 3 of them are derivatives of butyrophenone (haloperidol, bromperidol and benperidol), 2 are diphenylbutylpiperidine derivatives (pimozide and penfluridol) and 2 are derivatives of phenothiazine (promazine) and thiazepine (quetiapine), respectively. All reagents were purchased from Sigma-Aldrich (St. Louis, MO, USA) and were used without further purification. All of the experiments, apart from cholinesterase inhibition and oxidative hemolysis inhibition, were conducted using the tested compounds at concentrations equaling their therapeutic plasma concentrations.

#### 4.1.2. Materials

The following reagents were used to perform enzymatic reactions: 0.1 mol/L phosphate buffer pH = 7.0 and pH = 8.0 (disodium phosphate and monosodium phosphate (J.T. Baker, Center Valley, PA, USA)); a stock aqueous solution of acetylthiocholine iodide (ATC; 21.67 mg/mL) (Sigma-Aldrich, St. Louis, MO, USA); and a stock aqueous solution of butyrylthiocholine iodide (BTC; 20.50 mg/mL) (Sigma-Aldrich, St. Louis, MO, USA); a stock solution of 5,5′- dithiobisnitrobenzoic acid (DTNB; 0.01 mol/L) (Sigma-Aldrich, St. Louis, MO, USA) was prepared in phosphate buffer at pH = 7.0. All solutions were stored in aliquots at a temperature of −30 °C and before each experiment they were restored at 37 °C for 15 min. To determine kinetic parameters and the type of inhibition, decreasing concentrations of ATC and BTC were used (1:2–1:20).

Aβ42 aggregation studies were performed using recombinant human β-amyloid (1–42; ultrapure) purchased from Sigma-Aldrich (St. Louis, MO, USA). Solutions of were stored in aliquots at a temperature of −30 °C and were restored at room temperature before each experiment. A solution of Thioflavin T (ThT; Sigma-Aldrich, St. Louis, MO, USA) was prepared in phosphate buffer at pH = 7.4 (disodium phosphate, monosodium phosphate (J.T. Baker, Center Valley, PA, USA)), stock concentration was 0.03 mol/L.

Reagents used to assess the antioxidant potential of antipsychotic drugs: 0.9% NaCl (0.15 mol/L) (Chempur, Poland); the Triton X-100 was obtained from Polish Chemical Reagents (Gliwice, Poland); potassium hexacyanoferrate (II) (K_4_[Fe(CN)_6_]) and 2,2′-azobis(2-methylpropionamidine) dihydrochloride (AAPH) were purchased from Sigma-Aldrich (St. Louis, MO, USA).

#### 4.1.3. Cell Culturing

Human umbilical vein endothelial cells (HUVEC) were purchased from Lonza (Clonetics, Basel, Switzerland), and cultured according to the manufacturer’s guidelines. The reagents for HUVECs included: EGM-2–medium + bullet kit (Lonza, Clonetics, Italy), HEPES buffered saline solution (Lonza, Clonetics, Italy) and Accutase (Sigma-Aldrich, St. Louis, MO, USA). Cell viability was assessed using WST-1 assay (Takara, Takara Bio Europe, Saint-Germain-en-Laye, France).

Astrocytes were purchased from Life Technologies (Thermo Fisher Scientific, Waltham, MA, USA). The Gibco Human Astrocytes Kit contains normal human cells and Gibco Astrocyte Medium Kit consisting of base medium (DMEM), N-2 Supplement, and OneShot Fetal Bovine Serum (FBS) (Thermo Fisher Scientific, Waltham, MA, USA). Cells were cultured according to the manufacturer’s guidelines using vessels (75 cm^2^) coated with Geltrex matrix (Thermo Fisher Scientific, Waltham, MA, USA). Dulbecco’s phosphate-buffered saline (DPBS) with calcium and magnesium ions (Thermo Fisher Scientific, Waltham, MA, USA) was used to rinse culture vessels. The astrocytes were detached from the plate using Accutase (Sigma-Aldrich, St. Louis, MO, USA).

#### 4.1.4. Preparation of Biological Material

The blood was obtained from healthy donors from the Voivodal Specialized Hospital in Łódź, Poland (Wojewódzki Specjalistyczny Szpital im. Dr W. Biegańskiego w Łodzi). The blood was collected into vacuum tubes containing sodium citrate (0.109 mol/L; 3.2%). Erythrocytes were separated from plasma by centrifugation (3000× *g*, 10 min, 20 °C) with a Micro 22 R centrifuge (Hettich Zentrifugen, Tuttlingen, Germany). Human erythrocytes and plasma were frozen separately and stored at a temperature of −30 °C for up to 2 months. Before the experiments, the erythrocytes or plasma were thawed at 37 °C for 15 min and used to determine AChE or BuChE activity, respectively. The studies on the biological material were approved by the Bioethics Committee of the Medical University of Lodz (RNN/278/19/KE).

### 4.2. Methods

#### 4.2.1. Cholinesterase Inhibition

The activity of both cholinesterases (AChE and BuChE) were assessed spectrophotometrically according to the Ellman’s method with some modifications by Worek et al. [95,96]. This test development included experimental determination of the optimal testing conditions. For all measurements, 37 °C was used as the best temperature for enzymatic determinations in material of human origin. In modified Ellman’s method the wavelength is changed from 412 nm to 436 nm. This made it possible to avoid high hemoglobin absorption at λ = 412 nm. The measurement at λ = 436 nm reduced the colored indicator TNB^−^ (3-carboxy-4-nitrobenzenethiolate anion) absorption to 80% and the hemoglobin absorption to 25%.

The experiments were performed using a Cecil CE 2021 spectrophotometer (CECIL Instruments Limited, Cambridge, UK) with circulating thermostated water (37 °C) in Semi-Micro cuvettes (Medlab Products, Raszyn, Poland). The solution of plasma 200-fold diluted with 0.9% NaCl and solution of hemolyzed erythrocytes diluted 400-fold with 0.9% were incubated (37 °C, 15 min) with 5 μL DTNB (0.01 mol/L) and 10 μL tested compound at a wider range of concentration than the therapeutic range in order to assess the overall effect of the compound on human esterases (AChE and BuChE). The controls without antipsychotics, containing only DTNB and diluted plasma or diluted solution of hemolyzed erythrocytes, were prepared. The reaction was started by adding 5 μL substrate (ATC or BTC; 0.75 μmol/mL). The final volume of a sample was 500 μL. The absorbance was measured at λ = 436 nm for 3 min continuously using the Data Stream CE 3000 5.0 computer program (Cecil Instruments Ltd., Cambridge, UK). The maximal velocity of the reaction was determined on the basis of changes in absorbance over time.

In order to validate the research method control, tests were carried out for both AChE and BuChE. Based on the obtained data, the coefficients of variance (CV) were determined CV_AChE_ = 4.24%; CV_BuChE_ = 7.10%. The obtained results are included in Supplementary Materials (Table S3, Supplementary Materials).

#### 4.2.2. Kinetic Parameters of Enzymatic Reaction Estimation

The experiments were carried out using the substrate: ATC or BTC (0.75 μmol/mL) in decreasing concentrations (2-, 3-, 5-, 10- or 20-fold) and inhibitors in concentrations equal to their IC_50_. The kinetic parameters for donepezil, rivastigmine, quetiapine and promazine were estimated from three individual experiments performed on three different biological materials. The absorbance was recorded at λ = 436 nm using a CECIL 2021 spectrophotometer (Cecil Instruments Ltd., Cambridge, UK) with a thermostatic water flow (temperature 37 °C).

#### 4.2.3. Potential Synergism between Antipsychotics and AChEIs towards the Inhibition of Human ChE

Potential synergistic effects between antipsychotics and donepezil/rivastigmine on ChE inhibition were performed using the modified Ellman’s method [95,96]. The samples (v = 470 μL) of 200-fold diluted plasma (BuChE inhibition) or 400-fold diluted solution of hemolyzed erythrocytes (AChE inhibition) were preincubated with 5 μL DTNB (0.01 mol/L) and a mixture (v = 20 μL) of donepezil or rivastigmine and antipsychotic drug for 15 min. Afterwards, a substrate (BTC or ATC, respectively, at the final concentration of 0.75 μmol/mL), was added. The concentration of donepezil was between 0.01 and 100 nmol/L for AChE inhibition, and between 0.2 and 100 μmol/L for BuChE measurements. The concentration of rivastigmine was 5–100 μmol/L for AChE inhibition, and 0.05–5 μmol/L for BuChE measurements. In turn, the concentrations of antipsychotics were constant in every measurement, and were chosen on the basis of estimated therapeutic plasma concentrations.

#### 4.2.4. Beta-Amyloid Aggregation Studies

Aβ42 peptide was dissolved in DMSO and left to hydrate for a few minutes. The solution was centrifuged at 10,000 rpm for 5 min at 4 °C to separate any precipitated material. The solution at a concentration of 220 μmol/L was stored in small aliquots at a temperature of −30 °C and before each experiment it was restored at 37 °C for 15 min. Test samples containing Aβ42 solution at the final concentration of 20 μmol/L was incubated with antipsychotics at concentrations equaling their TPC and 1/2 × TPC (v = 10 µL). The final volume of the test samples was 100 μL. The positive control constituted a sample of Aβ42 peptide, whereas the negative control contained tannic acid at the final concentrations of 0.1 µmol/L and 10 µmol/L. The fibrillation reaction was set up by addition of thioflavin ThT dye at a concentration of 5 µmol/L. The fluorescence intensity was measured at room temperature with Ex/Em = 440 nm/484 nm (Synergy H1; BioTek, Winooski, VT, USA). The measurements of fluorescence intensity expressed as relative fluorescence units (RFU) were taken at 10, 30, 60 and 90 min and then after 24 and 48 h. The fluorescence intensity measured for control equaled 100% of Aβ aggregation and it was used to estimate the inhibitory properties of the tested antipsychotics.

Conditions to perform the Aβ aggregation test were experimentally selected based on the literature [97,98,99,100,101,102,103,104]. This allowed us to obtain better results for the parameters of variability and better reproducibility of the obtained results. Control tests were carried out in order to validate the research method. The obtained results, which allowed us to determine the CV (CV = 2.49%), are included in Supplementary Materials (Table S4, Supplementary Materials).

#### 4.2.5. ROS-Induced RBC Hemolysis

The blood samples were centrifuged (3000 rpm, 10 min, 20 °C, Micro 22 R centrifuge, Hettich Zentrifugen, Tuttlingen, Germany) to separate erythrocytes from the plasma and washed three times with 0.9% saline (NaCl). The hemolytic activity was assessed in human erythrocytes according to the method described by Baldivia et al. [105]. The erythrocytes were suspended in 0.9% saline. As control of basal hemolysis, erythrocytes were incubated with 0.9% NaCl. For total hemolysis control erythrocytes were incubated with 2.0% *v*/*v* Triton X-100. Incubation with Triton X-100 and K_4_[Fe(CN)_6_] (at concentration 50 g/L) was performed to control methemoglobin (MetHb) formation. Moreover, an antioxidant control containing ascorbic acid (AA) at a concentration of 10 µg/mL was prepared, while AAPH at a concentration of 50 mmol/L was an oxidizing agent. Antipsychotics at concentrations equaling their maximum therapeutic plasma concentrations (TPC_max_), as well as 5-fold higher (5 × TPC_max_) and 5-fold lower than TPC_max_ (1/5 × TPCmax), and the oxidizing agent AAPH at a concentration of 50 mmol/L, were added to 2% RBC suspension. Controls and tested samples were incubated at 37 °C. The measurements of absorbance were made after 5 h and 24 h at λ = 540 nm to determine the release of hemoglobin from damaged erythrocytes and at λ = 630 nm to determine the oxidation of hemoglobin to methemoglobin [105,106]. For each measurement, 0.5 mL samples were taken from incubated Eppendorf tubes and centrifuged (3000 rpm, 10 min). The absorbance of supernatants was measured using a CECIL 2021 spectrophotometer (CECIL Instruments Limited, Cambridge, UK). The percentage of hemolysis was calculated with the following formula:

Hemolysis (%) = (A_sample_/A_total hemolysis_) × 100% (1)

where A_sample_—absorbance of the test sample; A_total hemolysis_—the absorbance of the reference sample with 2% Triton X solution, corresponding to complete hemolysis.

#### 4.2.6. Antioxidative Potential of Antipsychotics in Cell Culture Model

WST-1 assay (Takara, Takara Bio Europe, Saint-Germain-en-Laye, France) was used to assess the effects of antipsychotics on the growth of HUVEC and astrocytes. The experiments were conducted as described previously [107]. HUVEC were seeded at a density of 7500 cells, and astrocytes at 5000 cells per well on 96-well microplates and cultured for 24 h followed by treatment with compounds at ½ × TPC_max_ and TPC_max_ or pure medium as a control (v = 100 μL) for another 24 h (37 °C, 5% CO_2_). Subsequently, the cells were washed with culture medium (100 μL) and WST-1 reagent diluted in medium (10 μL of reagent + 90 μL of medium) was added. The plates were incubated at 37 °C in 5% CO_2_ for another 90 min for HUVEC and 60 min for astrocytes. The absorbance was read at 450 nm using a microplate reader (iMARK, Bio-Rad, Hercules, CA, USA). The cells viability was expressed as a percentage of the control samples which constituted 100% viability.

Moreover, the WST-1 assay was used to evaluate potential protective effects of antipsychotics on the growth of HUVEC and astrocytes under conditions of AAPH-induced oxidative stress. The experimental conditions were the same as in viability studies. AA at the final concentration of 10 μg/mL was used as an antioxidant control. After reaching the confluence, the antipsychotics were added at concentrations equaling ½ × TPC_max_ and TPC_max_. After 1 h incubation under standard conditions, 50 μL AAPH was added to the wells containing tested compounds or AA. The AAPH concentration was chosen on the basis of preliminary experiments—17.5 mmol/L in HUVEC experiments, and 15.0 mmol/L in the case of astrocytes.

#### 4.2.7. Data Analysis

A statistical analysis was conducted with a commercially available package (Statistica 12.0, StatSoft, Krakow, Poland) and GraphPad Prism 8 (La Jolla, San Diego, CA, USA). The results were expressed as the mean ± standard deviation (SD).

The IC_50_ value, defined as drug concentration that inhibits 50% of the activity of an enzyme, was calculated on the basis of the equation y = a × ln (x) + b, linear regression (y = a × x + b) or quadratic equations (y = a × x^2^ + b × x + c). AChE SI (selectivity index) was calculated by using the following formula: SI = IC_50_ of BuChE/IC_50_ of AChE. However, BuChE SI was defined as IC_50_ of AChE/IC_50_ of BuChE. Maximal velocity (*V*_max_) and the Michaelis constant (*K*_m_) were calculated with the use of linear regression—according to the Hanes–Woolf plot.

The median–effect principle described by Chou et al. [36] allowed us to investigate multiple drug effects on ChEs. The method involves plotting effective dose curves for each drug and binary mixtures thereof at different doses. Automatic simulation of synergism and antagonism at all doses or effect levels is possible by computer software, based on algorithms. Calculation of the combination index (CI) and isobologram analyses enable to quantitatively determine drug interactions, where CI < 1, =1 and >1 indicate synergism, additive effect and antagonism, respectively. CompuSyn software (ComboSyn, Paramus, NJ, USA; http://www.combosyn.com; Accessed on 5 January 2022) was used to perform all calculations. The software also allowed us to display the dose–effect curve, the median–effect plot and the dose-reduction index (DRI) plot [36].

## 5. Conclusions

Herein, we reported the potential inhibitory properties of antipsychotic drugs (haloperidol, bromperidol, benperidol, penfluridol, pimozide, quetiapine and promazine) in relation to AChE and BuChE, as well as the potential synergism between antipsychotics and donepezil and rivastigmine towards ChEs. Our research makes clinical implications for the validity of combined therapies in the course of AD and their output efficacy. In our study, we also showed for the first time that most of the tested antipsychotics—the use of which is not limited to AD therapies—may inhibit the early stages of Aβ monomer bonding and even late Aβ aggregation, associated with the linking of large fibrils. The protective effect of the medications also manifests itself in a reported decrease in vulnerability of neural cells to oxidative stress. This in vitro study, partly supported by mathematical model analyses and in silico results, may become a basis for an in vivo follow-up clinical research to further elucidate the impact of the presented interactions between the tested group of drugs and antipsychotics.

## Figures and Tables

**Table 1:** Effects of donepezil, rivastigmine, promazine and quetiapine on human erythrocyte acetylcholinesterase and plasma butyrylcholinesterase activity. Results of IC_50_ are presented as mean ± SD, *n* = 6–9.

Compound	IC_50_ (µmol/L)		SI		References
	AChE	BuChE	AChE	BuChE	
Donepezil	0.025 ± 0.004	12.81 ± 1.52	512.4	0.002	experimental data
Donepezil—reference values according to the literature	0.323 ± 0.126	12.80 ± 0.70	39.6	0.025	[28]
	0.02 ± 0.0004	4.60 ± 0.28	230.0	0.004	[29]
	0.035 ± 0.003	2.32 ± 0.10	66.3	0.015	[30]
Rivastigmine	64.29 ± 2.97	0.95 ± 0.09	0.015	67.67	experimental data
Rivastigmine—reference values according to the literature	4.76 ± 0.11	0.24 ± 0.02	0.05	19.83	[28]
	3.12 ± 0.46	0.38 ± 0.02	0.12	8.21	[31]
	8.10 ± 0.33	3.60 ± 0.15	0.44	2.25	[32]
	56.1 ± 1.4	66.3 ± 5.3	1.18	0.85	[33]
Promazine	>2 *	0.19 ± 0.02	0.05 *	19 *	experimental data
Quetiapine	>100 *	6.08 ± 1.63	0.009 *	113 *	experimental data

*—theoretical values calculated on the basis of extrapolated plots for promazine and quetiapine towards AChE and BuChE. SI—selectivity index: for AChE, SI is defined as IC_50_ BuChE/IC_50_ AChE affinity ratio, for BuChE as IC_50_ AChE/IC_50_ BuChE.

**Table 2:** Kinetic parameters of enzymatic reactions: *K*_m_, *V*_max_—kinetic parameters for pure enzyme; *K*_m(i)_, *V*_max(i)—_kinetic parameters of tested compounds (donepezil, rivastigmine at concentrations equal to their IC_50_ values, and promazine, quetiapine at concentrations equal to their IC_50_ and ½ IC_50_ values).

	AChE	BuChE
	Donepezil [IC_50_]	
*K*_m_ [µmol/mL]	0.084 ± 0.017	0.085 ± 0.007
*K*_m(i)_ [µmol/mL]	0.180 ± 0.054	0.149 ± 0.009
*V*_max_ [A/min]	0.247 ± 0.019	0.245 ± 0.069
*V*_max(i)_ [A/min]	0.125 ± 0.027	0.098 ± 0.033
	Rivastigmine [IC_50_]	
*K*_m_ [µmol/mL]	0.099 ± 0.059	0.066 ± 0.015
*K*_m(i)_ [µmol/mL]	0.200 ± 0.084	0.062 ± 0.030
*V*_max_ [A/min]	0.277 ± 0.022	0.236 ± 0.023
*V*_max(i)_ [A/min]	0.119 ± 0.023	0.122 ± 0.003
	Promazine [IC_50_]	
*K*_m_ [µmol/mL]	n.d.	0.110 ± 0.013
*K*_m(i)_ [µmol/mL]	n.d.	0.600 ± 0.224
*V*_max_ [A/min]	n.d.	0.234 ± 0.012
*V*_max(i)_ [A/min]	n.d.	0.144 ± 0.036
	Quetiapine [IC_50_]	
*K*_m_ [µmol/mL]	n.d.	0.077 ± 0.004
*K*_m(i)_ [µmol/mL]	n.d.	0.097 ± 0.033
*V*_max_ [A/min]	n.d.	0.169 ± 0.046
*V*_max(i)_ [A/min]	n.d.	0.138 ± 0.058
	Promazine [½ IC_50_]	
*K*_m_ [µmol/mL]	n.d.	0.106 ± 0.031
*K*_m(i)_ [µmol/mL]	n.d.	0.465 ± 0.170
*V*_max_ [A/min]	n.d.	0.251 ± 0.021
*V*_max(i)_ [A/min]	n.d.	0.192 ± 0.059
	Quetiapine [½ IC_50_]	
*K*_m_ [µmol/mL]	n.d.	0.106 ± 0.031
*K*_m(i)_ [µmol/mL]	n.d.	0.100 ± 0.017
*V*_max_ [A/min]	n.d.	0.251 ± 0.021
*V*_max(i)_ [A/min]	n.d.	0.239 ± 0.026

Results are presented as mean values ± SD of two independent experiments, conducted in duplicates or triplicates; n.d.—not determined (insufficient inhibition of AChE to determine IC_50_ value).

**Table 3:** Effects of antipsychotics on the anti-AChE and anti-BuChE properties of donepezil. Results are presented as mean ± SD, *n* = 6–9; * *p* < 0.05 vs. IC_50_ of pure donepezil.

Donepezil				
	IC_50_ AChE		IC_50_ BuChE	
Donepezil	25.58 ± 4.56 [nmol/L]		12.81 ± 1.52 [μmol/L]	
	**IC_50_ Binary** **Mixtures** **[nmol/L]**	**Effect**	**IC_50_ Binary** **Mixtures** **[nmol/L]**	**Effect**
Haloperidol (0.07 μmol/L)	25.08 ± 2.44	-	7.85 ± 0.53	↓ 38.7%
Bromperidol (0.06 μmol/L)	24.33 ± 1.43	↓ 4.9%	5.88 ± 0.49 *	↓ 54.1%
Benperidol (0.03 μmol/L)	28.66 ± 0.17	↑ 12.1%	5.39 ± 0.41 *	↓ 57.9%
Penfluridol (0.05 μmol/L)	25.37 ± 0.47	-	11.56 ± 0.86	↓ 9.8%
Pimozide (0.03 μmol/L)	20.74 ± 2.26	↓ 18.9%	7.19 ± 0.19 *	↓ 43.8%
Quetiapine (0.91 μmol/L)	34.32 ± 1.78	↑ 34.2%	6.18 ± 0.12 *	↓ 51.8%
Promazine (0.15 μmol/L)	29.90 ± 2.24	↑ 16.9%	4.51 ± 0.19 *	↓ 64.8%

**Table 4:** Effects of antipsychotics on the anti-AChE and anti-BuChE properties of rivastigmine. Results are presented as mean ± SD, *n* = 6–9. * *p* ˂ 0.05 vs. IC_50_ of pure rivastigmine.

Rivastigmine				
	IC_50_ AChE		IC_50_ BuChE	
Rivastigmine	64.29 ± 2.97 [μmol/L]		0.95 ± 0.09 [μmol/L]	
	**IC_50_ Binary** **Mixtures** **[nmol/L]**	**Effect**	**IC_50_ Binary** **Mixtures** **[nmol/L]**	**Effect**
Haloperidol (0.07 μmol/L)	49.79 ± 2.37 *	↓ 22.6%	1.08 ± 0.08	↑ 13.6%
Bromperidol (0.06 μmol/L)	33.26 ± 1.39 *	↓ 48.3%	0.91 ± 0.03	-
Benperidol (0.03 μmol/L)	39.70 ± 0.24 *	↓ 38.2%	1.01 ± 0.09	↑ 5.6%
Penfluridol (0.05 μmol/L)	64.51 ± 2.74	-	1.18 ± 0.07 *	↑ 23.7%
Pimozide (0.03 μmol/L)	32.81 ± 2.99 *	↓ 51.0%	0.76 ± 0.06	↓ 20.3%
Quetiapine (0.91 μmol/L)	34.75 ± 2.16 *	↓ 45.9%	1.01 ± 0.07	↑ 6.4%
Promazine (0.15 μmol/L)	44.20 ± 3.46 *	↓ 31.2%	1.24 ± 0.04 *	↑ 30.0%

## Data Availability

The datasets generated during the current study are available from the corresponding author on reasonable request.

## Supplementary Materials

The following supporting information can be downloaded at: https://www.mdpi.com/article/10.3390/ijms23094621/s1.
