# Supplementary material for: The Influence of Selected Antipsychotic Drugs on Biochemical Aspects of Alzheimer’s Disease
Source: Int J Mol Sci. 2022 Apr 21;23(9):4621. doi: 10.3390/ijms23094621 (PMC9102502; doi:10.3390/ijms23094621)
Supplement: Supplementary file 1 [file ijms-23-04621-s001.zip › ijms-1656295-supplementary.pdf]

Supplementary Material

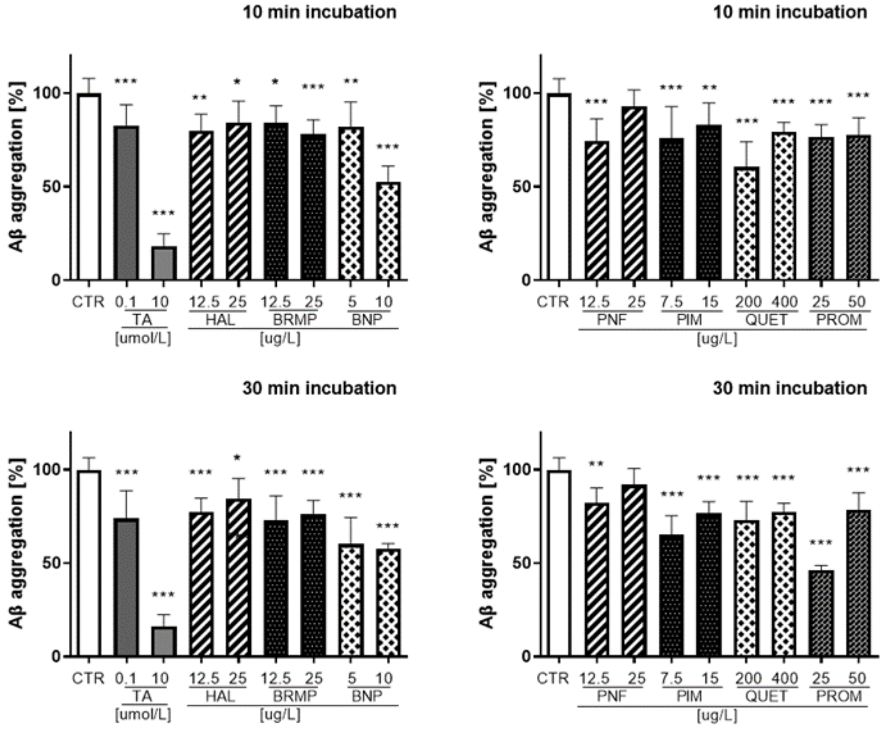

Figure S1. The effects of tested antipsychotics at ½ TPC and TPC on Aβ aggregation depending on the fibrillation time: 10 min, 30 min. Tannic acid at a concentration of 0.1 μmol/L and 10 μmol/L (170.12 μg/L and 17012 μg/L, respectively) was used as an inhibitor of Aβ aggregation. The results are presented as mean ± SD of three independent experiments conducted in triplicates. *$p < 0.05$, **$p < 0.01$, ***$p < 0.001$ versus control.

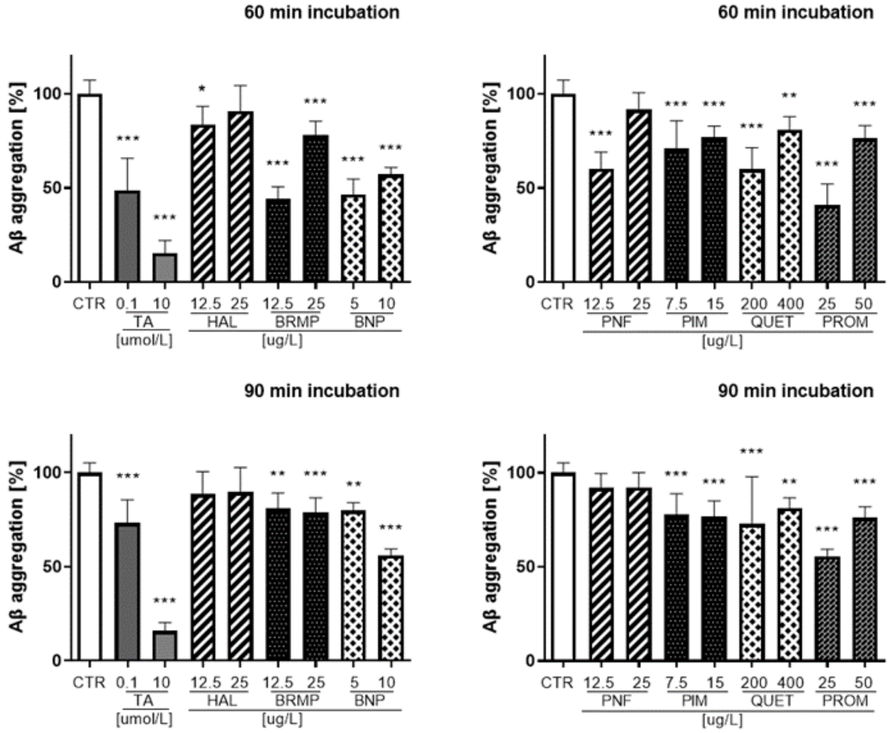

Figure S2. The effects of tested antipsychotics at ½ TPC and TPC on Aβ aggregation depending on the fibrillation time: 60 min, 90 min. Tannic acid at a concentration of 0.1 μmol/L and 10 μmol/L (170.12 μg/L and 17012 μg/L, respectively) was used as an inhibitor of Aβ aggregation. The results are presented as mean ± SD of three independent experiments conducted in triplicates. *$p < 0.05$, **$p < 0.01$, ***$p < 0.001$ versus control.

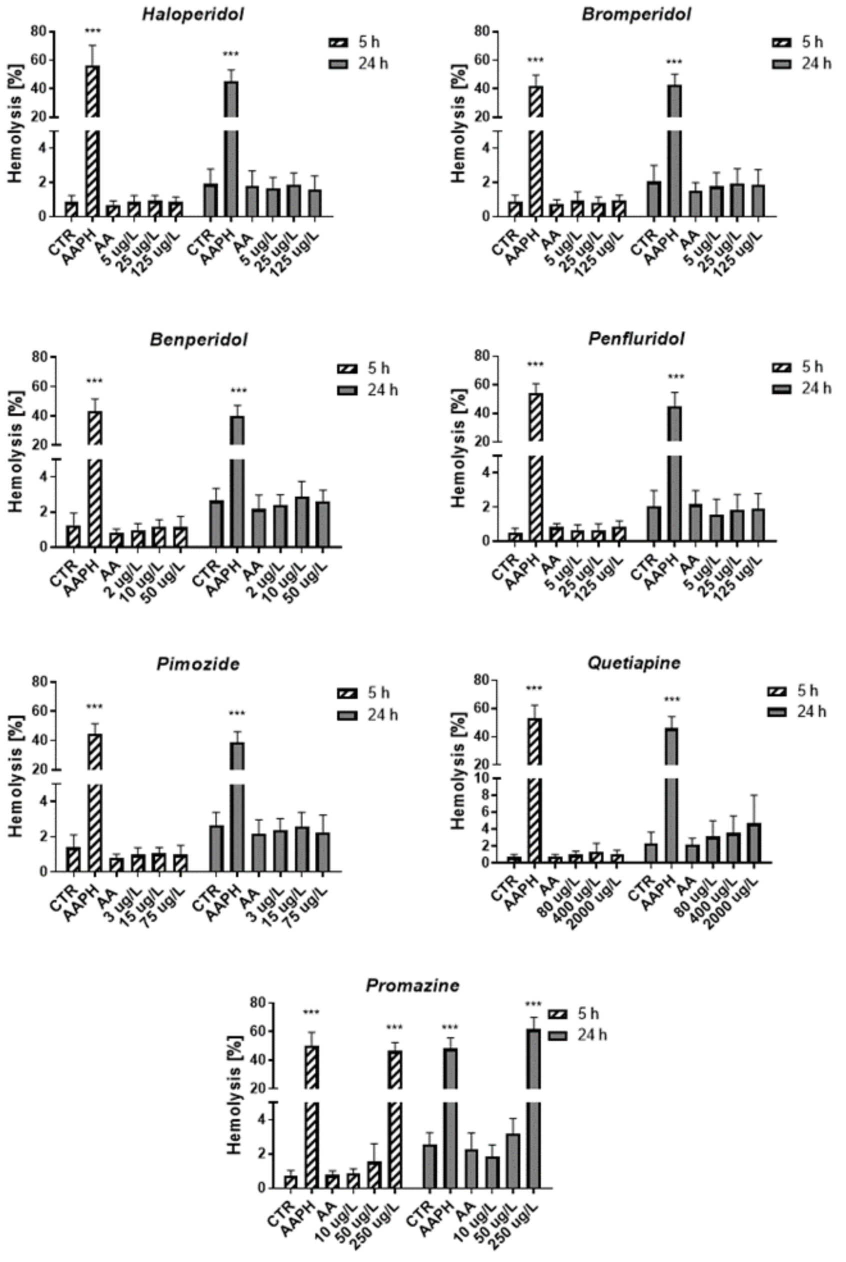

Figure S3. Effect of tested antipsychotics at 1/5 × TPC_{max}, TPC_{max} and 5 × TPC_{max} on erythrocyte hemolysis measured after 5 h and 24 h of incubation. AAPH was used at the concentration of 50 mmol/L and AA at the concentration of 10 μg/mL. The results are presented as mean ± SD, n = 6–12, ***$p < 0.001$ versus control.

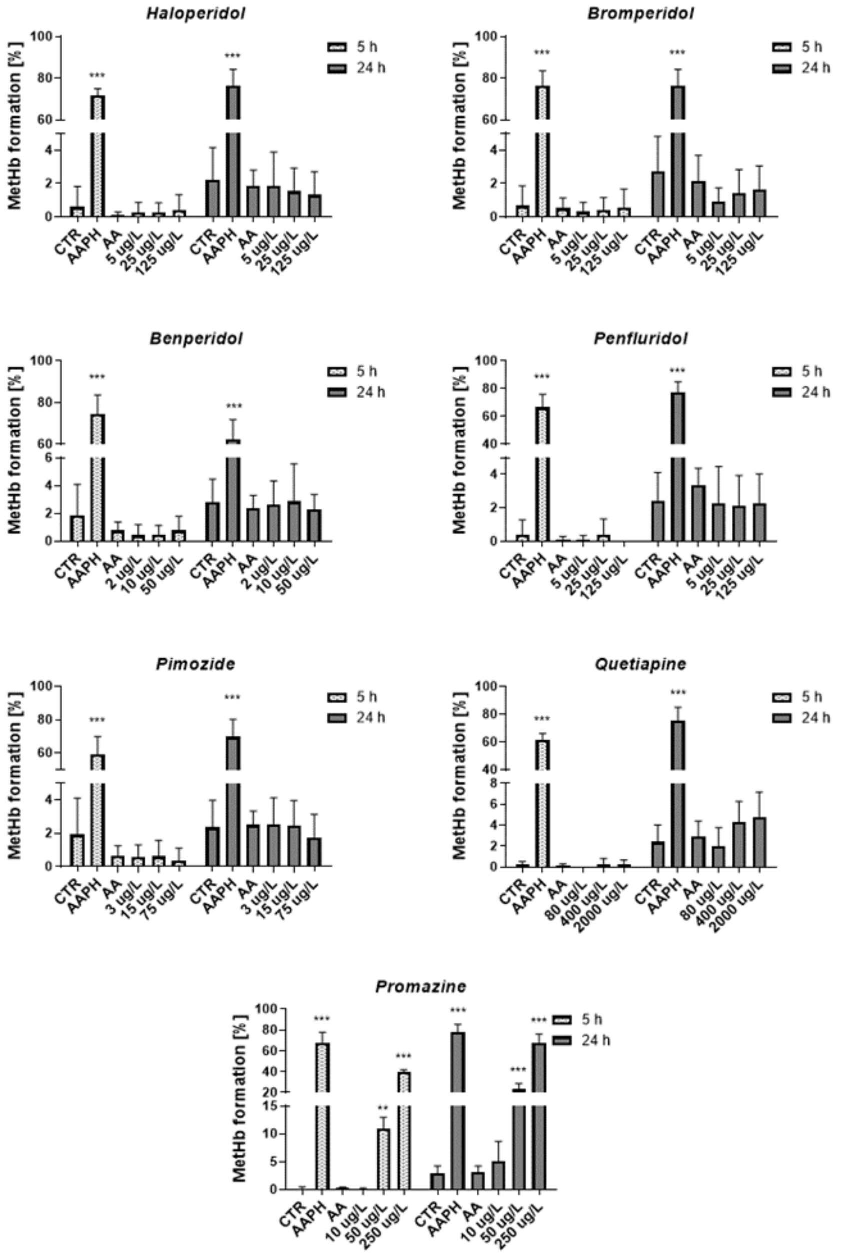

Figure S4. Effect of tested antipsychotics at 1/5 × TPC_{max}, TPC_{max} and 5 × TPC_{max} on methemoglobin formation measured after 5 h and 24 h of incubation. AAPH was used at the concentration of 50 mmol/L and AA at the concentration of 10 μg/mL. The results are presented as mean ± SD, n = 4–12, $^{**}p < 0.01$, $^{***}p < 0.001$ versus control.

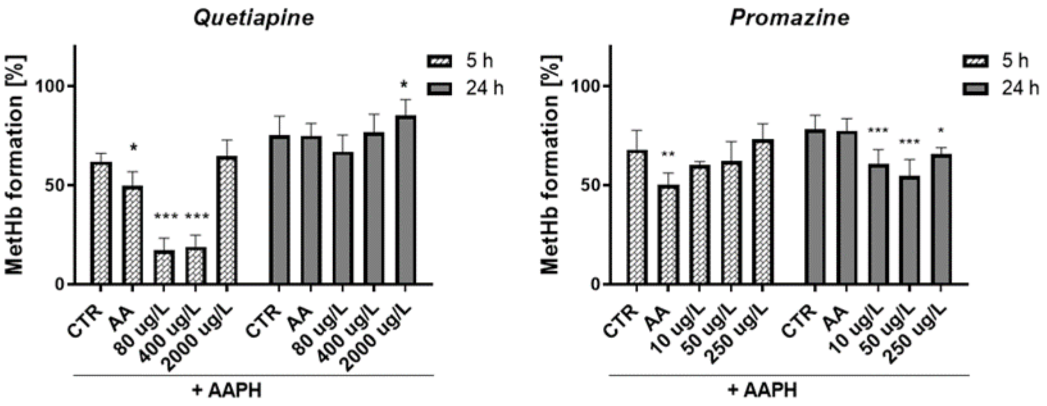

Figure S5. Effect of quetiapine and promazine at 1/5 × TPC_{max}, TPC_{max} and 5 × TPC_{max} with AAPH on methemoglobin formation measured after 5 h and 24 h of incubation. AAPH was used at the concentration of 50 mmol/L and AA at the concentration of 10 μg/mL. The results are presented as mean ± SD, n = 6–12, *$p < 0.05$, **$p < 0.01$, ***$p < 0.001$ versus control.

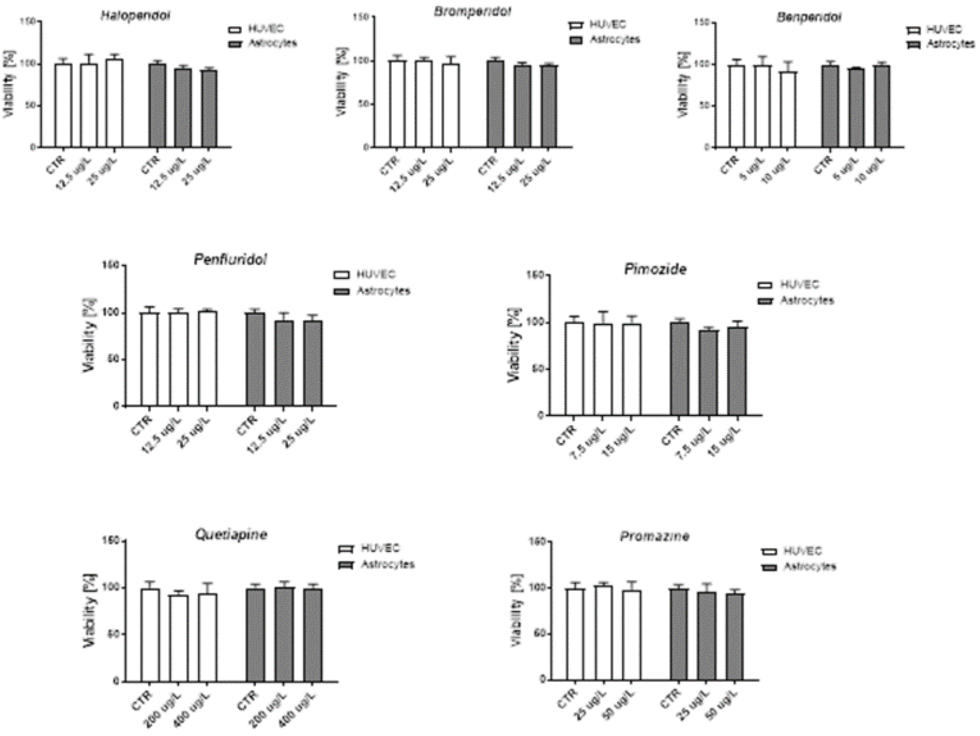

Figure S6. Effects of studied antipsychotics on the viability of two human cell lines, human umbilical vein endothelial cells (HUVEC) and astrocytes assessed by the WST-1 test. Compounds were tested at two concentrations corresponding to ½ x therapeutic plasma concentration (TPC) and TPC. The results are presented as mean ± SD, n = 8–9. None of the studied compounds contributed to the significant decrease in cells' viability ($p > 0.05$).

Table S1. The values of the CI determined by means ComboSyn software for binary mixtures with variable concentration of donepezil (range 0.005–0.1 µmol/L for AChE and 2–100 µmol/L for BuChE) and constant concentration of antipsychotic (at their TPC_{max}).

**AChE**	**CI Values for Tested Antipsychotics**						
**Donepezil [µmol/L]**	**HAL [0.07 µmol/L]**	**BRMP [0.06 µmol/L]**	**BNP [0.03 µmol/L]**	**PNF [0.05 µmol/L]**	**PIM [0.03 µmol/L]**	**KWET [0.91 µmol/L]**	**PROM [0.15 µmol/L]**
0.005	>2.50	0.07	0.83	0.79	0.50	1.83	1.34
0.01	>2.50	0.78	1.09	0.80	0.63	1.50	1.04
0.02	>2.50	0.81	1.04	0.93	0.62	1.23	1.12
0.025	>2.50	0.73	1.03	0.90	0.65	1.24	1.06
0.05	0.37	0.81	1.02	0.89	0.65	1.25	0.97
0.075	0.16	0.70	1.17	1.05	0.66	1.22	0.88
0.1	0.09	0.80	1.24	0.90	0.68	1.24	0.80
**BuChE**	**CI Values for Tested Antipsychotics**						
2	0.71	0.43	0.40	1.21	0.57	0.64	0.61
5	0.67	0.52	0.50	0.98	0.55	0.64	0.56
10	0.79	0.57	0.57	1.04	0.78	0.71	0.56
20	0.77	0.70	0.60	1.03	0.76	0.75	0.64
50	0.84	0,79	0.69	1.14	0.91	0.85	0.78
100	0.87	0.89	0.84	1.21	0.79	0.95	0.81

CI—(Combination Index), where CI < 1, =1, and >1 indicate synergism, additive effect, and antagonism, respectively.

Table S2. The values of the CI determined by means ComboSyn software for binary mixtures with variable concentration of donepezil (range 5–100 µmol/L for AChE and 0.05–5 µmol/L for BuChE) and constant concentration of antipsychotic (at their TPC_{max}).

**AChE**	**CI Values for Yested Antipsychotics**						
**Rivastigmine [µmol/L]**	**HAL [0.07 µmol/L]**	**BRMP [0.06 µmol/L]**	**BNP [0.03 µmol/L]**	**PNF [0.05 µmol/L]**	**PIM [0.03 µmol/L]**	**KWET [0.91 µmol/L]**	**PROM [0.15 µmol/L]**
5	0.39	0.32	0.31	>2.50	0.26	0.25	0.52
10	0.55	0.38	0.44	0.75	0.37	0.37	0.66
15	0.60	0.46	0.48	0.73	0.38	0.39	0.62
25	0.70	0.50	0.57	0.92	0.47	0.49	0.68
40	0.71	0.53	0.62	0.97	0.56	0.40	0.66
50	0.79	0.49	0.63	097	0.57	0.57	0.72
75	0.86	0.55	0.66	1.06	0.62	0.61	0.72
100	0.82	0.60	0.65	1.06	0.65	0.59	0.81
**BuChE**	**CI values for tested antipsychotics**						
0.05	0.61	0.18	0.27	0.64	> 2.50	2.24	0.66
0.1	0.44	0.30	0.32	0.68	> 2.50	1.42	0.67
0.25	0.67	0.57	0.56	0.79	> 2.50	1.02	0.85
0.5	0.87	0.74	0.91	1.01	> 2.50	1.03	1.04
0.75	1.06	0.86	0.95	1.08	> 2.50	1.04	1.14
1	1.03	0.92	1.00	1.13	> 2.50	1.00	1.30
2.5	1.19	0.96	1.08	1.28	> 2.50	1.01	1.78
5	1.22	0.96	1.00	1.17	> 2.50	1.10	2.27

CI—(Combination Index), where CI < 1, =1, and >1 indicate synergism, additive effect, and antagonism, respectively.

Table S3. The values of AChE and BuChE inhibition measurements made for the controls (CTR) to validate the Ellman's method with some modifications.

AChE	Measurement 1 [A/min]	Measurement 2 [A/min]	The Average of Reaction Velocity [A/min]	SD	CV [%]
CTR1	0.181	0.187	0.180	0.008	4.24
CTR2	0.172	0.172			
CTR3	0.186	0.191			
CTR4	0.177	0.172			
**BuChE**	**Measurement 1 [A/min]**	**Measurement 2 [A/min]**	**The Average of Reaction Velocity [A/min]**	**SD**	**CV [%]**
CTR1	0.165	0.155	0.163	0.012	7.10
CTR2	0.163	0.169			
CTR3	0.178	0.140			
CTR4	0.165	0.172			

The results presented for four samples conducted in duplicates, with the mean value, standard deviation (SD) and the coefficient of variance (CV) [%].

Table S4. The values of fluorescence intensity expressed as relative fluorescence units (RFU) for the controls (CTR) to validate the method used for β-amyloid aggregation studies.

	Measurement 1 [RFU]	Measurement 2 [RFU]	The Sverage [RFU]	SD	CV [%]
CTR1	5152	5230	5113	127	2.49
CTR2	5117	5325			
CTR3	4958	5091			
CTR4	5080	4947			

The results presented for four samples conducted in duplicates, with the mean value, standard deviation (SD) and the coefficient of variance (CV) [%].
